# Genome editing techniques in plants: a comprehensive review and future prospects toward zero hunger

**DOI:** 10.1080/21645698.2021.2021724

**Published:** 2022-02-09

**Authors:** Naglaa A. Abdallah, Aladdin Hamwieh, Khaled Radwan, Nourhan Fouad, Channapatna Prakash

**Affiliations:** a Department of Genetics Faculty of Agriculture, Cairo University, Cairo, Egypt; b National Biotechnology Network of Expertise, ASRT, Egypt; c Department of Biotechnology, International Centre for Agricultural Research in the Dry Areas (ICARDA), Giza, Egypt; d Department of Biotechnology, Agricultural Genetic Engineering Research Institute (AGERI), ARC, Giza, Egypt; e Tuskegee University, Tuskegee, AL, USA

**Keywords:** CRISPR/Cas, base editing, prime editing, food security, crop improvenet

## Abstract

Promoting sustainable agriculture and improving nutrition are the main United Nation’s sustainable development goals by 2030. New technologies are required to achieve zero hunger, and genome editing technology is the most promising one. In the last decade, genome editing (GE) using the CRISPR/Cas system has attracted researchers as a safer and easy tool for genome editing in several living organisms. GE has revolutionized the field of agriculture by improving biotic and abiotic stresses and yield improvement. GE technologies were developed fast lately to avoid the obstacles that face GM crops. GE technology, depending on site directed nuclease (SDN), is divided into three categories according to the modification methods. Developing transgenic-free edited plants without introducing foreign DNA meet the acceptance and regulatory ratification of several countries. There are several ongoing efforts from different countries that are rapidly expanding to adopt the current technological innovations. This review summarizes the different GE technologies and their application as a way to help in ending hunger.

## Introduction

Eradicating hunger and malnutrition are great significant challenges that many countries work to solve. Global climatic changes and economic downturns due to the COVID-19 pandemic are hindering the world from achieving the Sustainable Development Goals (SDGs), with a final goal of eliminating hunger by 2030. In general, the main factors affecting food security include limited availability of land and water resources, high rates of population growth, and limitation of the domestic food systems to respond to demand (availability, nutrition, access). According to the World Food Programme (www.wfp.org), more than 135 million people are suffering from acute hunger and the number is expected to surpass 840 million by 2030. In addition, unpredictable global climatic changes, and the emergence of resistant pathogens are severe threats to global food security. To ensure food security, all stakeholders should work together to establish a stringent program by improving the uptake of agricultural and food security research into policy and practice to solve food insecurity. To eliminate poverty, we need to increase food production sustainably and ensure that food is well distributed. Several challenges face agricultural production, including sustainably and maintaining yield, grain quality, improving the nutritional value, and acquiring resistance against biotic and abiotic stresses. Scientists have the great responsibility toward achieving zero hunger by the year 2030, improving nutrition, and promoting sustainable agriculture.

One way to ensure food security is through developing sustainable crops that are adopted to changing environments. Crops were developed during the Green Revolution (started in the mid-1940s and attributed to Norman Borlaug, the Nobel Peace Prize winner, 1970) with high-yielding varieties that produced an increased grain per area planted. However, conventional breeding techniques are not sufficient to meet these challenges and achieve food security. The new precision biotechnologies, involving genetically modified (GM) and genome editing (GE) technology, could speed up breeding, and the integration of ‘omics’ technology makes that possible. New Breeding Techniques (NBT) need exhaustive knowledge of the genome of the target species to enable the development of a new crop carrying the target trait(s). The pangenome, the entire gene set of all strains of a species, provides useful informations concerning the genomic variations in the gene pool for a given cultivated species. Pangenome represents genes present in all strains (core genome) and that which is only present in some strains (variable or accessory genome). Super-pangenome offers good opportunities for crop improvement by studying the complete genomic variation range of a given genus. Super-pangenome includes wild species and their application for crop improvement.^1^ Structural variations in the given genomes play an essential role in plant genetics. They include phenotyping-based selection, marker-based breeding, genomics-assisted breeding (GAB), genetically modified organisms (GMO), and genome editing (GE). Moreover, developing superior varieties requires prerequisite identifications of markers/loci/genes that are connected with the trait of interest.^2^ It is worth to mention that GE technology emerged in 2003 to improve various crop characteristics and does not involve using genes derived from different organisms other than the species of interest.

Recently, foods improved using GE technology have received considerable attention concerning its safety. GE is a precise modification that has no or minor changes to traditional crops modified breeding. In the coming decade, it is expected that GE will replace GM as it improves various crop characteristics through a higher success rate and lack of external gene insertion. Instead, the target genes are identified, cut, and modified in very precise ways.

GE could be achieved through enzymes that are collectively called site-directed nucleases (SDN) directed by DNA binding domain or by RNA molecules to bind to the genome’s target site. Thus, they affect only specific endogenous sequences. Protein-binding systems include meganucleases (MegaNs), zinc finger nuclease (ZFN), transcription activator-like effector nucleases (TALENs), while the RNA binding molecules have clustered regularly interspaced short palindromic repeats (CRISPR)/CRISPR-associated protein system (CAS). CRISPR/Cas9 system requires short guide sequence RNA (sgRNA) to direct Cas9 nuclease to cleave the double-stranded DNA target site complementary to the sgRNA. CRISPR-Cas system is the most commonly used system of eukaryotic genomes, as it has several advantages, including precise manipulation, effectiveness, ease of use, inexpensiveness, and allowing multiple genome manipulation.^3–5^

## Evolution of CRISPR-Cas Systems

### First Generation of CRISPR-Cas Systems

#### CRISPR/Cas9 System

**Figure 1.**
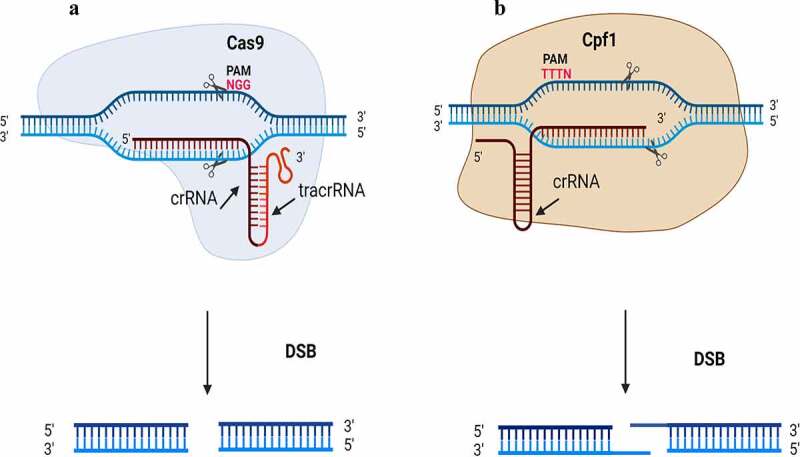
CRISPR-Cas9 versus CRISPR-Cpf1; Cas9, crRNA and tracrRNA represent a fused single-guide RNA, while Cpf1 needs crRNA only. The PAM sequence is trinucleotide 5′-NGG-3′for Cas9 while is 5ʹ-TTN for Cpf1. Cas9 cleavage dsDNA with blunt ends ends 3 nt upstream of the PAM site, while Cpf1 cleaves in a 5ʹ overhang sticky ends 18–23 bases apart from the PAM. Complementary sequence to the target DNA is linked to 5′ crRNA end of Cas9 and to 3′ ends of crRNA.

The CRISPR system was first identified as an adaptive defensive mechanism that confers resistance to foreign genetic elements in bacteria and archaea, and the CRISPR-Cas system was first used for eukaryote genome editing 2013. The programmed Cas9 binds to a small guide RNA (sgRNA), the CRISPR/Cas9 endonuclease first scans the genomic DNA and binds upstream of the G-rich protospacer adjacent motif (NGG-PAM) sequences (Fig. 1a). Cas9 generate a double-strand breakage (DSBs) in the targeted DNA and sgRNA guides Cas9 to the target site to form the RNA–DNA duplex followed by an adjacent protospacer motif (PAM) of the genome.^6–8^ Moreover, CRISPR/Cas9 genome editing was extensively implemented for trait improvement of different economically important crops.^9–12^ Cas9 nickase (nCas9) is to be mutated in one of the catalytic residues of the nuclease domains (H840A in HNH and D10A in RuvC) in a way that can only generate single-strand breaks (SSBs), a process that can reduce off-target effects of CRISPR/Cas system^13^ In addition, nCas9 increase editing specificity and improve targeting ranges for gene, epigenetic, and base editing.^14,15^

#### CRISPR/ Cpf1 (Cas12a) System

The Cpf1 (previously named Cas12a) is a class-II, Type V CRISPR derived from *Prevotella* and *Francisella*, and it is significantly more accurate and efficient in genome manipulation. Cpf1 is a small size monomeric protein, processes pre-crRNA into mature crRNA without a tracrRNA with more working efficiency. It recognizes T-rich PAM sequence, either 5ʹ -TTTN-3ʹ or 5ʹ -TTTV-3ʹ sequence (V = A, C or G), bind downstream of the motif and form struggled ends of DNA with 4–8 nucleotide-long^15–17^ (Fig. 1b). The overhangs with sequence complementarity into a genome would provide more efficient genomic insertions and offer more flexibility in base editing and epigenetic modulation.^18^

### New Generation of CRISPR-Cas Systems

#### Base Editing System

Base editing (BE) is the precise modification of a single-nucleotide variants at a certain loci in the target DNA (nuclear or organellar) or RNA of a living cell using a catalytically impaired Cas nuclease that is fused to a nucleotide deaminase. The recently developed DNA BEs include adenine BEs (ABEs), cytosine BEs (CBEs), dual-base editors, and organellar BEs.

**Figure 2.**
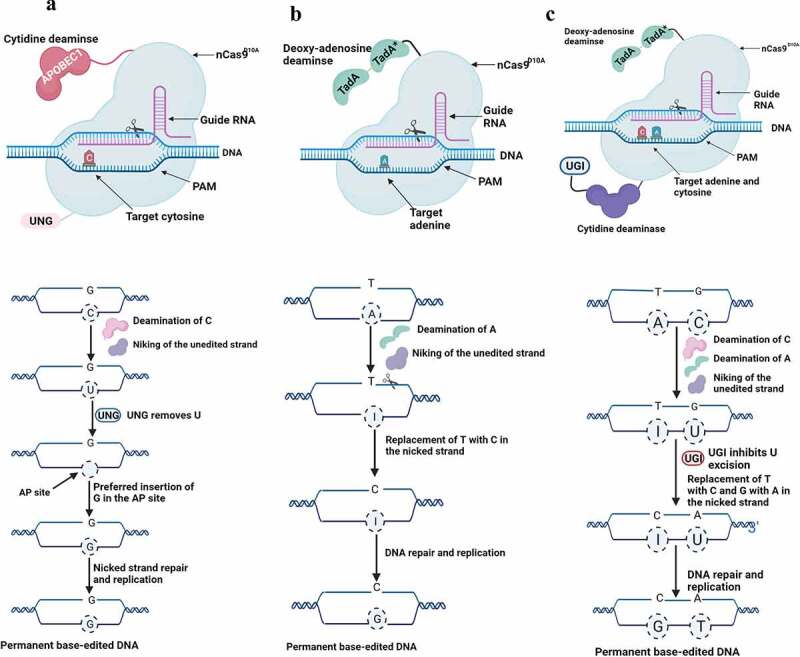
Nuclear base editing mechanism: a) Cytosine base editing (CBE) consisting of cytidine deaminase, nCas9 and UGI. The cytosine deaminase converts the desired “C” to “U,” the resulted mismatch can be corrected by base editing repair or cellular mismatch repair. The nick produced by nCas9 in the guanidine “G”-containing unedited DNA strand remove the “G” by cellular mismatch repair using uracil as a template for repair leading to the targeted “T•A.” Uracil is removed from the DNA by uracil DNA N-glycosylase, thus, reverting to the original “C•G” binding. The rate of obtaining “T•A” is enhanced through the increase of UGI protein b) Adenine base editing (ABE) contains a heterodimeric deaminase linked to nCas9. ABE is composed of wild-type TadA monomer and engineered TadA (TadA*) monomer. The target base “A” is deaminated to inosine (i) leading to converting “A•T” pair to an “I•T” bp. Inosine pairs with “C” during the replication. c) Dual-base editor converts “C-to-T” and “A-to-G.” The complex nucleoprotein is composed of nCas9, adenosine deaminase, cytidine deaminase and UGI. In the dual-base editing, deamination of “C” and “A” is performed by cytidine and adenosine deaminase, respectively. The dual base editors has the same mode of action of that of both CBE and ABE.

***CBEs*** were the first developed DNA BEs to enable C.G to T.A transitions using Cas9 nickase fused to cytidine deaminase and uracil glycosylase inhibitor (UGI). The engineered sgRNA–CBE binds to the target DNA generating a ssDNA R-loop. Thus, the non-target ssDNA becomes accessible to CBE cytidine deaminase causing hydrolytic deamination of an exposed cytosine (C). The cellular mismatch of the deaminated base repair results in changing C-to-T. Uracil (U) base excision repair (BER) either keeps the original base pair or gives rise to an indel. UGI subverts uracil base excision repair and increases the probability of switching C-to-T (Fig. 2a). Interestingly, CBEs have been applied in different plant species, including Arabidopsis,^19^ rice,^20^ wheat,^21^ maize,^22^ tomato and potato,^23^ cotton,^24^ soybean,^25^ strawberry,^26^ apple and pear.^27–29^ While the activity window within the single-stranded DNA in the R loop that has access to the cytidine deaminase is ~4–10 nucleotides long for the majority of the Cas9-based Bes, it ranges from 8 to 13 next to the T-rich PAM motif (counted as 1) for Cas12a-based CBE. Nevertheless, developing Cas12a nickase is complicated and was only designed for base editing mammalian cells 28, 29.

***ABEs*** were developed through combining adenine deaminase and nCas9 and were used to convert an A-T base pair to a G-C base pair using tRNA adenosine deaminase (TadA) variant, which works on a ssDNA substrate.^30^ A TadA works as a dimer to catalyze deamination, a heterodimeric protein consisting of the wild-type TadA non-catalytic monomer. An engineered catalytic monomer (TadA*) was developed and fused with nCas9 to convert A to G (Fig. 2b). ABEs were applied to develop rice,^31–35^ wheat,^33^ Arabidopsis and *Brassica napus*,^36^ *Nicotiana benthamiana*,^37^ poplar,^38^ and moss.^39^

***Dual-base editors*** were developed through fusing cytidine and adenosine deaminases into Cas protein (Fig. 2c) to manage C-to-T and A-to-G substitutions into a genomic region of interest. Nevertheless, individual CBE and ABE operations and dual-base editors share the same mode of action; deamination of C and A which is carried out by cytidine and adenosine deaminase, respectively.

#### Genome Editing for Plant Organelles

The genome of the mitochondria and chloroplasts encode essential genes for cellular respiration and photosynthesis, respectively. Gene editing in plant organelles was developed to efficiently promote point mutagenesis in chloroplasts and mitochondria using a protein-based system such as ZFN and TALEN. Although the CRISPR–Cas-derived techniques are highly effective for nuclear genome editing, they are not applicable for editing organellar DNA. It is difficult to deliver or to express both gRNA and the Cas enzyme to organelles. Mitochondria-Targeted genome editing was used in plants to disrupt the cytoplasmic male sterility (CMS)-associated genes using mitochondria-TALENs.^40,41^ Also, genome editing could be used to induce point mutation in the 16S rRNA gene in the chloroplast genome to enhance antibiotic resistance.^42,43^

**Figure 3.**
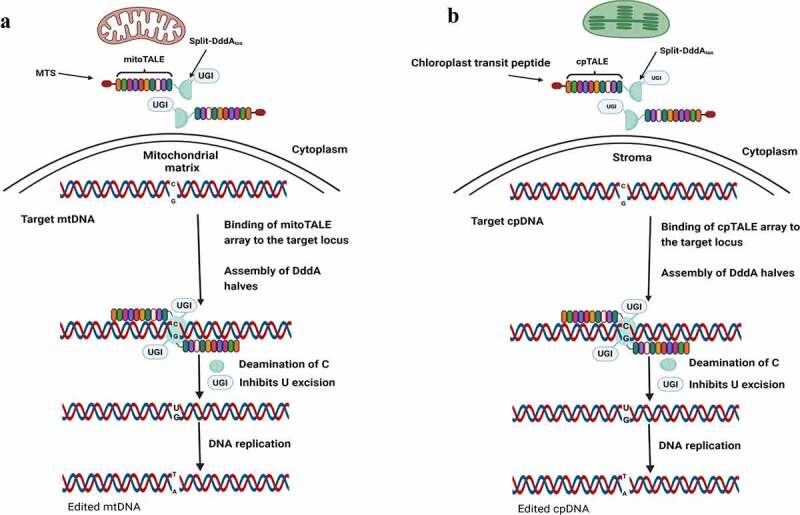
Mechanism of Organelle base editing involves TALE array, a DddA cytidine deaminase (DdCBE), a UGI to catalyze cytosine deamination, inducing “C-to-T” conversions and mitochondrial targeting signal (MTS) for editing mitochondrial DNA (a) or Chloroplast transit peptide (CTP) for editing Chloroplast DNA (b). The MTS transports the two halves of TALE into the mitochondrial matrix. The chloroplast transit peptide target the TALE array to chloroplast matrix. Two TALE arrays bind to the desired DNA sequence bringing the two DssA inactive halves into proximity. After reconstitution of active DddAtox, it deaminate “C” in the double-stranded DNA.

Organelle base editing-based systems were developed based on mitochondrial targeting signal (MTS) or Chloroplast transit peptide (CTP), a TALE array, a DddA cytidine deaminase (DdCBE) as well as a UGI to catalyze cytosine deamination, inducing C-to-T conversions (Fig. 3). The mitochondrial genome editing system depends on the introduced genes’ nuclear gene expression, followed by transporting the expressed proteins to the mitochondria. While, in chloroplast base editing, a biolistic DNA gun is used for direct delivery. DdCBE with DNA binding domains of TALEN system was recently used for editing chloroplast and mitochondrial genome of lettuce, rapeseed, *Arabidopsis*, and rice.^43–45^

#### CRISPR/Cas13

**Figure 4.**
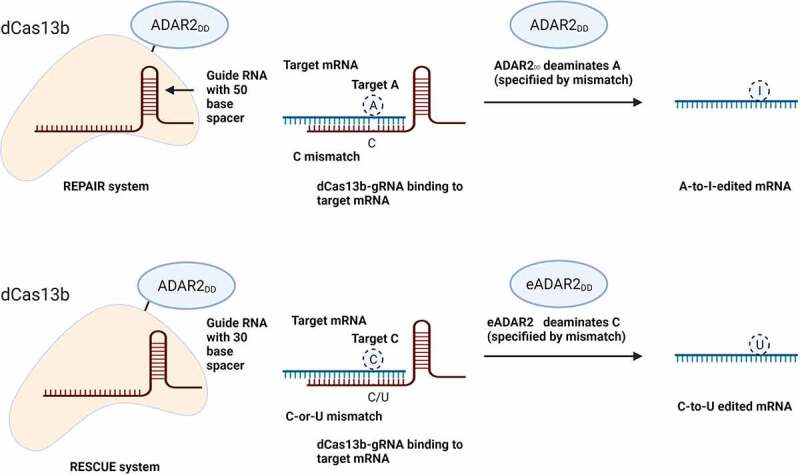
Mechanism of base editing in RNA. A) In the REPAIR system, “A-to-I” editing is using dCas13 fused to ADAR2. REPAIR use 50-nucleotide RNA with a 50-nucleotide mRNA-gRNA duplex. “A–C” mismatch in the RNA–gRNA duplex determines the target A. RESCUE system editing “C-to-U.” The optimum results are to be achieved with a gRNA of 30-nucleotide spacer. The target “C” is specified by an induced “C–C” or “C–U” mismatch in the mRNA–gRNA duplex.

The Cas13a was first identified in 2016^46^ as an RNA-targeting CRISPR enzyme. Cas13b is class 2 – type VI, identified as RNA-guided RNA-targeting CRISPR–Cas system. It is used for precise target and cleavage ssRNA^47^ without PAM requirements.^48^ Cas13 was modified to adapt to RNA base editing (RBE) by using dead Cas13b (dCas13b) for either “A-to-I” editing or “C-to-U” editing. Fusing the adenosine deaminases to dCas13b deaminates A, the target A induced “A–C” mismatch in the mRNA–gRNA duplex result in editing “A-to-I.”^49^ While fusing engineered ADAR2 (acting as cytosine deaminase) to dCas13b results in editing “C-to-U” by an induced “C–C” or “C–U” mismatch in the mRNA–gRNA duplex (Fig. 4).

#### Prime Editing System (PE)

**Figure 5.**
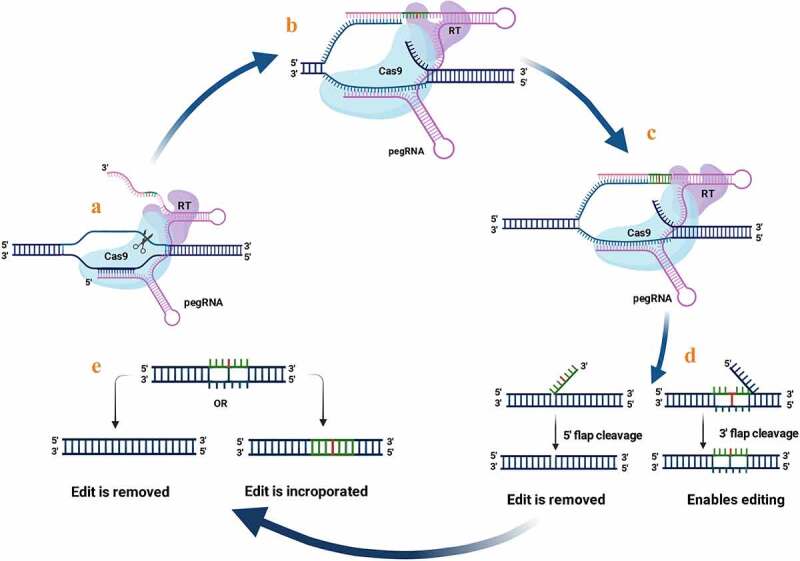
Prime editing mechanism: a) Nicking the desired DNA sequence at the PAM strand by the fusion protein, b) the exposed 3ʹ-hydroxyl group prime the reverse transcription of the RT template of the pegRNA, c) reverse transcription, d) the branched intermediate form containing two flaps of DNA: a 3ʹ flap (containing the edited sequence), and a 5ʹ flap (containing the dispensable, unedited DNA sequence) followed by flap cleavage, and e) ligation and mismatch repair; either the incorporating edite strand or remove it.

**Figure 6.**
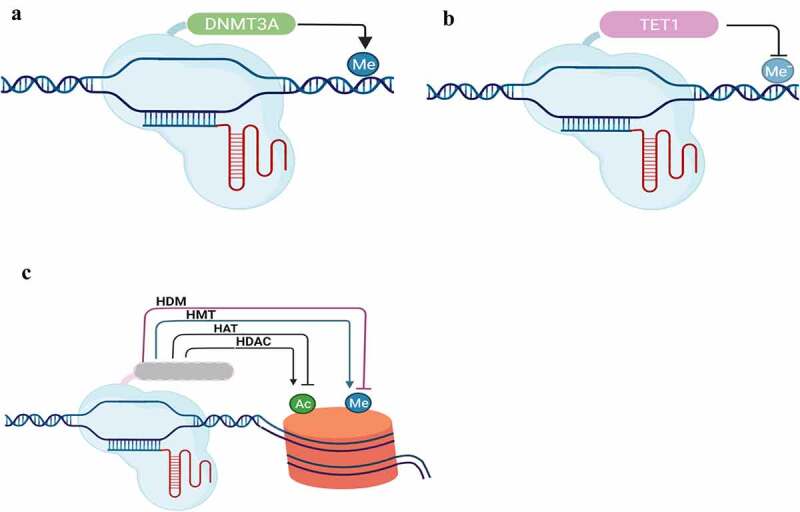
CRISPR/dCas9-based engineering of the epigenome. The techniques are based on using inactive-Cas (dCas) to allocate the desired protein to the target sequence. For DNA methylation, cytosine methylation could be used by linking dCas with DNMT3A or MQ1 (a), while demethylation of cytosine could be edited by linking Tet1 with dCas (b). Chromatin modifiers could be edited either by acetylation/deacetylation using HDAC/HAT or methylation/demethylation using HMT/HDM with dCas9.

PEs are multipurpose and precise genome editing tools that introduce all possible transition and transversion mutations and small indels. They use nCas9 that is fused to an engineered reverse transcriptase (RT) to write new genetic information into a specified DNA site. The RT is programmed with a prime editing gRNA (pegRNA) that specifies the target site and encodes the desired edit.^50,51^ PegRNA consists of an RT template, gRNA (crRNA), and a primer-binding site (PBS) (Fig. 5). The nCas9 nick the editing strand of the double-stranded DNA, and it is used for priming the reverse transcription of PBS on the pegRNA.^50^ Incorporating the edited DNA strand into the target DNA results in a heteroduplex DNA that contains only one edited strand. To resolve the heteroduplex, DNA repair machinery uses the edited strand as a template to copy the information from the edited strand to the complementary one. PEs led to a permanent incorporation of the preferred edit into the targeted region of the double-stranded DNA (Fig. 6).

#### Epigenome Editing System

Recent (CRISPR)/Cas-based epigenome editing (epi-editing) technologies site-specifically perform epigenetic modifications (methylases/demethylases) to endogenous DNA and histones, altering the chromatin structure and changing the accessibility of the transcriptional machinery, causing changes in gene expression. Histone methylation takes place at lysine and arginine residues.

The modifications of Epi-editing systems include histone acetylation using p300, histone demethylation using LSD1, cytosine methylation using MQ1 or DNMT3A, and cytosine demethylation using Tet1. In this system, dead Cas9 (dCas9) is fused with epigenetic effector domains to precise targeting promoter and enhancer regions to alter gene activation or repression. Epi-editing changes gene expression or cellular phenotype without alteration of DNA sequences and is inheritable in plants. They are used to de-methylate promoter to activate gene or to methylate promoter to deactivate it.

### Applications of Genome Editing in Agriculture

**Figure 7.**
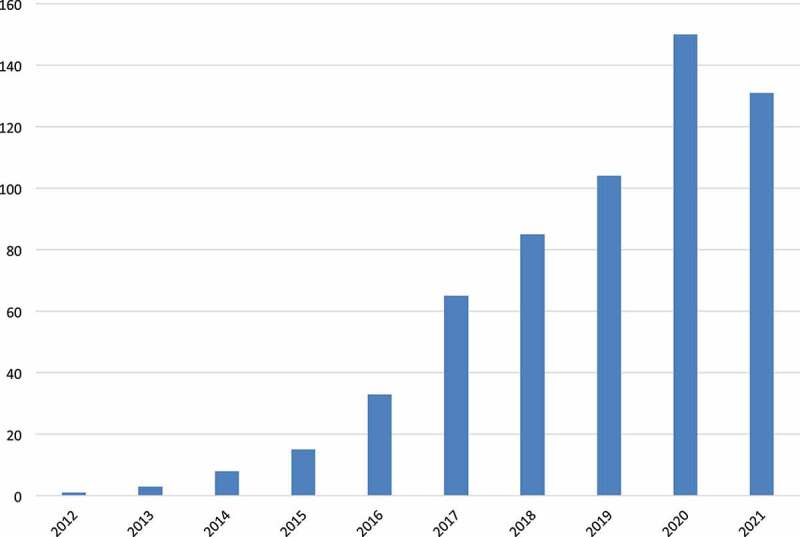
Number of CRISPR-based plant genome-editing publications over the last 10 years.

**Figure 8.**
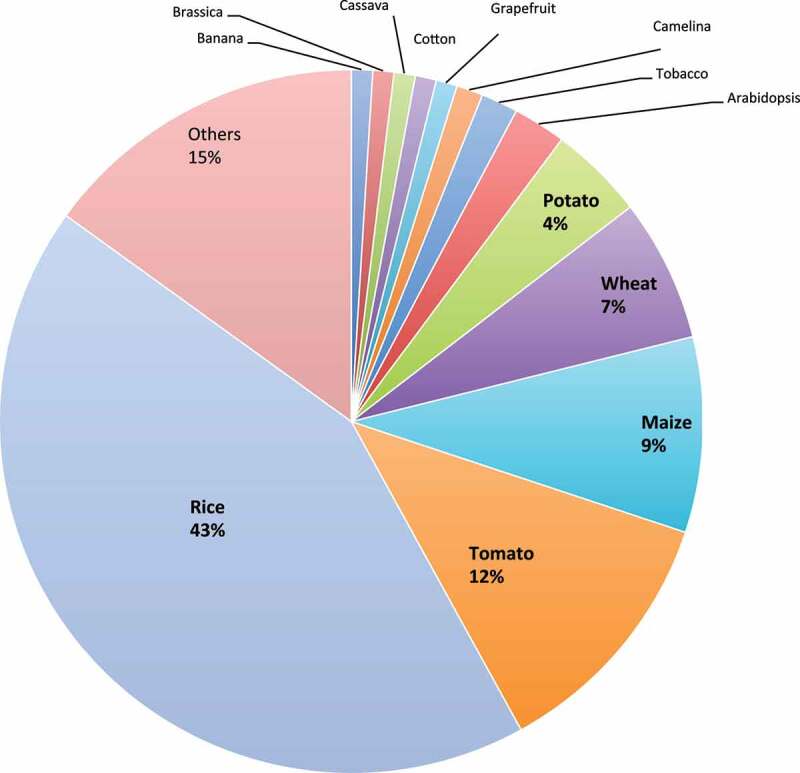
Percentage of major crops modified by CRISPR system with the aim of crop improvement.

CRISPR-based genome editing approach participated in different crop improvements, including abiotic (Table S1), and biotic stress management (Table S2) and breeding improvement (Table S3). The last decade witnessed increase in publications for the applications of this technology in different plants (Fig. 7). GE has been used to improve more than 40 crops, mainly rice, tomato, maize, wheat, and potato (Fig. 8).

#### Abiotic Stress Tolerance

Abiotic stresses are major threats to agricultural productivity and are anticipated to become more threatening in agricultural systems due to climate change. Recently, researchers targeted genome editing techniques for broadening resistance of crop tolerance, including drought, salinity, high temperature, and other climate change factors.

#### Salinity Tolerance

The area of salinization globally is increasing 10% annually, and it is expected by the year 2050 that more than 50% of arable lands worldwide will be salinized.^51^ In rice, the CRISPR/ Cas9 system was used to knock down the gene OsRR22 (encoding the rice type-B response).^21^ Moreover, CRISPR-Cas9 was used to mutate the salt and drought tolerance (*DST*) gene in indica rice cv. MTU1010. The mutated *dst* with 366 bp deletion showed enhanced leaf water retention due to its broadened-width leaves and the reduced stomatal density. Thus, *dst* mutant alleles could improve salt and drought tolerance as well as enhancing grain yield in rice.^52^

Moreover, in tomatoes, CRISPR/Cas9 was used to induce mutation to the *SlARF4* gene. Down-regulation of *SlARF4* promoted root development leading to higher soluble sugars and chlorophyll content under stress conditions. Thus, Mutated tomato plants successfully increased leaf relative water content and Abscisic acid (ABA) content and reduced stomatal conductance under normal and stressful conditions to tolerate osmotic and salt stresses^53^.

#### Drought Tolerance

Precise genomic DNA modification CRISPR‐Cas was used to introduce the native maize GOS2 promoter into the 5′‐untranslated region of the native *ARGOS8* gene. *ARGOS8* variants enhanced grain yield by five bushels per acre under flowering stress conditions. Nevertheless, it showed no yield loss under well‐watered conditions.^54^ Also, in tomato, the CRISPR/Cas9 system was utilized to generate slmapk3 mutants by knocking out SlMAPK3. Mutants showed up- or down-regulated expressions of drought stress-responsive genes (*SlLOX, SlGST, and SlDREB*), suggesting that knocking out SlMAPK3 protected cell membranes from oxidative damage and modulated transcription of stress-related genes.^55^

#### Thermotolerance Enhancement

The CRISPR-Cas9-mediated genome editing for heat tolerance was achieved by targeting the tomato’s SlAGAMOUS-LIKE 6 (*SIAGL6*) gene. Knocking out the SIAGL6 gene improved the fruit setting of tomatoes under heat stress.^56^ Moreover, CRISPR-Cas was used to disrupt OsMYB30 that belongs to B-Amylase (BMY) genes that regulate starch degradation and the accumulation of maltose. Thus cold-tolerant rice lines that are protecting against cold stress were generated^57^.

### Disease Resistance

Plant pathogens, viruses, fungi, and bacteria cause various damages to crop health, and reduce yield significantly. CRISPR/Cas system has been adapted to generate varieties that can resist pathogen attack based on alteration of plant immunity. CRISPR-Cas targets plant susceptibility (Su) genes that encode factors that support pathogen infection and decrease disease susceptibility. For controlling bacterial infection, knocking out *Xa13, OsSWEET13, OsSWEET13* by CRISPR/Cas9 enhanced resistance to bacterial blight in rice. While knocking down *TaMLO* and *TaEDR1* genes in wheat control the spread of powdery mildew.^58,59^ Moreover, CRISPR-Cas system was used to develop rice tungro virus resistance by disrupting the Su eIF4G in rice.^60^

Also, the CRISPR-Cas system was used to modify genes that facilitate pathogen growth in the host. In rice, mutating the fungal genes ALB1and RSY1 with CRISPR/Cas9 was used to control the spread of rice blast, while knocking out USTA and UvSLT2 has improved was used to prevent smut disease.^61^ While targeting viral genome mediated by CRISPR-Cas system was implemented to control geminivirus by disrupting viral replication, including Tomato Yellow Leaf Curl Virus (TYLCV) and Wheat Dwarf Virus (WDV) infection in tomato and barely targeting viral genome region directly (MP, CP, Rep, IR).^62,63^

### Increasing Yield and Quality

Grain yield is controlled through quantitative trait loci (QTLs) that affect grain number, weight, and size. CRISPR/Cas system was used to knockout plant genes that negatively regulate crop yields. In the rice, CRISPR/Cas9 was used to silence the expression of *OsGS3*, which has a negative impact on grain size and consequently on grain yield. While editing *OSGW* genes resulted in an increase in grain weight in rice, knockout *TaCKX2-D1* increased grain number^64^. Moreover, fruit size in tomato was improved by mutating *SICLV3* promoter and SIENO gene using the CRISPR/Cas9 system.^65^

### Nutritional Enhancement

Gene editing plays an important role in facing the malnourishment pandemic by increasing desirable nutritional metabolites, reducing anti-nutrients and altering the composition of macronutrients. Starch quality was improved by disrupting the two genes; *IbGBSSI* and *IbSBEII* in sweet potato^66^. Nutrient quality in bananas was improved by knocking down *LCYe* gene leading to increasing beta-carotene content.^67^ Disrupting the alpha-gliadin gene in wheat was used to lower gluten contents to reduce allergenicity^68^ Moreover, the oleic acid content was increased by editing *FAD2* gene in *Brassica napus* and peanut.^69^

Knockouts of the *SBEIIb* gene in rice decreased levels of amylopectin in favor of amylose in the rice endosperm^70^ In cassava, reduced starchy content was performed by knocking out two genes involved in amylose biosynthesis.^71^

CRISPR-Cas9 has also used to modify the starch contents in potato through knocking out of the *GBSS* gene in potato.^72^ Also, in strawberry, CRISPR/Cas9 was used to alter its sugar content through editing the gene *FvebZIPs1.1*.^26^

### Manipulating Plant Breeding

CRISPR/Cas system was applied to develop breeding materials through several methods such as controlling the development of male sterility, hybrid vigor, and self-incompatibility, and hybrid seed production is sometimes challenging to restrict self-pollination. Knocking down fertility genes such as *TMS5* in rice and *TaNP1* in wheat, using CRISPR/Cas9, developed male sterility.^69^

Knocking down Male sterility gene can be classified as either cytoplasmic male sterility (CMS) and genic male sterility (GMS), depending on the fertility gene source.^70^

Self-fertilization is important to enhance plant breeding. In potato, knocking out the S-RNase locus resulted in RNA degradation of the pollen tube and developed self-fertile lines.^71^

Haploid induction is essential for the breeder to develop double haploid and stabilize the genetic architecture of inbred lines. The CRISPR-Cas system participated in haploid induction in several plants such as wheat, maize, rice, and Arabidopsis. Knocking down the pollen-specific phospholipase gene in rice (*OsMTL*) resulted in spermatid chromatin fragmentation and haploid induction.^72^

### Regulatory Approaches to Genome Editing

GE could generate genetic variants at specific particular target sites that are indistinguishable from naturally evolved ones. The legislation and regulation of genome-edited plants in many countries are still evolving. Several countries have adapted their biosafety regulations based on this classification of variations induced by site-directed nuclease. Depending on the editing approach, three types of alterations could be distinguished.^73^ In the SDN-1, the DNA breakage introduces base-pair changes or insertions/deletions. Spontaneous repair is unguided repair and leads to gene silencing. SDN-2 requires a small DNA donor to target and repair the DNA break and generate a specific change. The donor carries the designed mutation(s) and flanking sequences complementary to both ends of the break. Homologous recombination between both the donor and the target cause DNA swap and allow introduction of the mutations. SDN-3 requires a prominent DNA donor of foreign origin to repair the damaged target sequence. The repair mechanism is also performed through the homologous recombination between the target and donor sequences. The donor DNA could contain part or complete gene and is considered transgenic.

The debate on the regulation of gene-edited crops is still going on. Certain countries regulate gene edited plants as GMOs based on the processed used for developing them, including EU union, Switzerland, and New Zealand. Other countries focus on the end-product and regulate them based on case-by-case rather than the process used for developing GE plants. This group includes the U.S., Canada, Argentina, Colombia, Brazil, Argentina, Chile Canada, and Japan. They authorize the registration of gene-edited crops if the end-product is free from any foreign DNA. While the discussion is ongoing for the third group of countries to follow the group to authorization authorize, such as the United Kingdom, Norway, Kenya, Nigeria, Paraguay, Uruguay, Norway, India, and Philippines.^72,74^

Argentina was the first to declare that GE crops are not be regulated under biosafety legislation if the plant do not contain foreign DNA, followed by Chile, Brazil, and Colombia. The decision taken by the biosafety authorities is based on case-by-case basis; plants containing new genetic materials will be regulated as a GMO.^75–79^ In contrast, USDA in the USA decided not to impose regulation on most plants produced by SDN-1 or SDN-2 once the CRISPR gene has been crossed out. GE *via* loss-of-function mutation only narrows the scope of improving plant yield and quality. It is worth to mention that the intragenics (within gene)/ cisgenics (whole gene) approaches are obviously more effective in leveraging genetic diversity and could be addressed to maximize the potential of agricultural improvements.

## Conclusion

Precise, efficient, and rapid genome editing technique is revolutionizing crop improvement by avoiding the genetic modification, gene disruption, and introduction unwanted genes (such as selectable marker genes). Genome editing can provide unprecedented solutions to food insecurity and malnutrition by developing higher-yielding, more nutritious crops and resilient to the impacts of biotic stresses and climate change.

GE mainly depends on the identification of target genes, delivery of CRISPR machinery to the right cells, selecting, and regenerating the right cells to crops. With the wealth of information obtained from genome studies and identifying genes related to crop improvement, GE techniques are providing innovative solution that could help address global food security by developing climate-smart crops with improved yield.

GE techniques that resulted in altering DNA/chromatin confirmation (epigenome editing), minimum but precise change in the genome (base editors), or precise insertion of short DNA fragments (prime editing) are promising candidates to bring about global regulation-overhaul, changes in the policy frameworks, and improved consumer acceptance.

## Supplementary Material

Supplemental Material
