# Supplementary material for: Genome editing techniques in plants: a comprehensive review and future prospects toward zero hunger
Source: GM Crops Food. 2022 Feb 9;12(2):601–15. doi: 10.1080/21645698.2021.2021724 (PMC9208631; doi:10.1080/21645698.2021.2021724)
Supplement: Supplemental Material [file KGMC_A_2021724_SM8963.zip › Table_S1.pdf]

Table S1: Summary of role of CRISPR system in abiotic stress improvement

Trait	Plant	Gene Function	Technique	Year	References
Cold tolerance	Rice	Cause sensitivity	CRISPR-Cas9	2017	Huang, X.; Zeng, X.; Li, J.; Zhao, D. Construction and analysis of tify1a and tify1b mutants in rice (Oryza sativa) based on CRISPR/Cas9 technology. J. Agric. Biotech. 2017, 25 (6), 1003-1012.
	Rice	Cause sensitivity	CRISPR-Cas9	2019	Huang, X.; Zeng, X.; Li, J.; Zhao, D. Construction and analysis of tify1a and tify1b mutants in rice (Oryza sativa) based on CRISPR/Cas9 technology. J. Agric. Biotech. 2017, 25 (6), 1003-1012.
	Rice	Cause sensitivity	CRISPR-Cas9	2019	Zeng, Y.; Wen, J.; Zhao, W.; Wang, Q.; Huang, W. Rational Improvement of Rice Yield and Cold Tolerance by Editing the Three Genes OsPIN5b, GS3, and OsMYB30 With the CRISPR–Cas9 System. Front. Plant Sci. 2020, 10, 1663.
Drough tolerance	Chickpea	Cause sensitivity	CRISPR-Cas9	2018	Badhan, S.; Ball, A. S.; Mantri, N. First Report of CRISPR/Cas9 Mediated DNAFree Editing of 4CL and RVE7 Genes in Chickpea Protoplasts. Int. J. Mol. Sci. 2021, 22 (1), 396.
	Maize	-	CRISPR/Cas9 SDN2	2018	Njuguna E, Coussens G, Aesaert S, Neyt P, Anami S, van Lijsebettens M. Modulation of energy homeostasis in maize and Arabidopsis to develop lines tolerant to drought, genotoxic and oxidative stresses. AF 2018. doi:10.21825/af.v30i2.8080.

Maize	-	CRISPR/Cas9 SDN4	2017	Shi J, Gao H, Wang H, Lafitte HR, Archibald RL, Yang M, et al. ARGOS8 variants generated by CRISPR-Cas9 improve maize grain yield under field drought stress conditions. Plant Biotechnol J. 2017;15:207–16. doi:10.1111/pbi.12603. 36
Maize	Cause sensitivity	CRISPR-Cas9	2017	Shi, J.; Gao, H.; Wang, H.; Lafitte, H. R.; Archibald, R. L.; Yang, M.; Hakimi, S. M.; Mo, H.; Habben, J. E. ARGOS 8 variants generated by CRISPR-Cas9 improve maize grain yield under field drought stress conditions. Plant Biotechnol. J. 2017, 15 (2), 20
Rice	Cause sensitivity	CRISPR-Cas9	2019	Liao, S.; Qin, X.; Luo, L.; Han, Y.; Wang, X.; Usman, B.; Nawaz, G.; Zhao, N.; Liu, Y.; Li, R. CRISPR/Cas9-Induced Mutagenesis of Semi-Rolled Leaf1, 2 Confers Curled Leaf Phenotype and Drought Tolerance by Influencing Protein Expression Patterns and ROS Scavenging in Rice (Oryza sativa L.). Agronomy 2019, 9 (11), 728.
Rice	Cause sensitivity	CRISPR-Cas9	2017	Lou, D.; Wang, H.; Liang, G.; Yu, D. OsSAPK2 confers abscisic acid sensitivity and tolerance to drought stress in rice. Front. Plant Sci. 2017, 8, 993.
Rice	Cause sensitivity	CRISPR-Cas9	2020	Zhang, Y.; Li, J.; Chen, S.; Ma, X.; Wei, H.; Chen, C.; Gao, N.; Zou, Y.; Kong, D.; Li, T. An APETALA2/ethylene responsive factor, OsEBP89 knockout enhances adaptation to direct-seeding on wet land and tolerance to drought stress in rice. Mol. Genet. Genomics 2020, 295 (4), 941-956.

	Soybean	-	CRISPR/Cas9 SDN1	2017	United States Department of Agriculture (USDA). 2017. https://www.aphis.usda.gov/biotechnology/downloads/reg_loi/17-219-01_air_inquiry.pdf. Accessed 25 Aug 2018.
	Wheat	-	CRISPR/Cas9 SDN1	2018	Kim D, Alptekin B, Budak H. CRISPR/Cas9 genome editing in wheat. Funct Integr Genomics. 2018;18:31–41. doi:10.1007/s10142-017-0572-x.
Heat tolerance	Tomato	Cause sensitivity	CRISPR-Cas9	2020	Klap, C.; Yeshayahou, E.; Bolger, A. M.; Arazi, T.; Gupta, S. K.; Shabtai, S.; Usadel, B.; Salts, Y.; Barg, R. Tomato facultative parthenocarpy results from Sl AGAMOUS-LIKE 6 loss of function. Plant Biotechnol. J. 2017, 15 (5), 634-647.
	Tomato	Cause sensitivity	CRISPR-Cas9	2021	Yu, W.; Wang, L.; Zhao, R.; Sheng, J.; Zhang, S.; Li, R.; Shen, L. Knockout of SlMAPK3 enhances tolerance to heat stress involving ROS homeostasis in tomato plants. BMC Plant Biol. 2019, 19 (1), 1-13.
Heavy metal tolerance	Rice	Cause sensitivity	CRISPR-Cas9	2020	Songmei, L.; Jie, J.; Yang, L.; Jun, M.; Shouling, X.; Yuanyuan, T.; Youfa, L.; Qingyao, S.; Jianzhong, H. Characterization and evaluation of OsLCT1 and OsNramp5 mutants generated through CRISPR/Cas9-mediated mutagenesis for breeding low Cd rice. Rice Sci. 2019, 26 (2), 88-97

	Rice	Cause sensitivity	CRISPR-Cas9	2020	Tang, L.; Mao, B.; Li, Y.; Lv, Q.; Zhang, L.; Chen, C.; He, H.; Wang, W.; Zeng, X.; Shao, Y. Knockout of OsNramp5 using the CRISPR/Cas9 system produces low Cdaccumulating indica rice without compromising yield. Sci. Rep. 2017, 7 (1), 1-12.
	Rice	Cause sensitivity	CRISPR-Cas9	2019	YANG, C.-H.; ZHANG, Y.; HUANG, C.-F. Reduction in cadmium accumulation in japonica rice grains by CRISPR/Cas9-mediated editing of OsNRAMP5. J. Integr. Agric. 2019, 18 (3), 688-697.
	Canola	-	ODM	2015	Gocal GFW, Schöpke C, Beetham PR. Oligo-Mediated Targeted Gene Editing. In: Advances in New Technology for Targeted Modification of Plant Genomes; 2015. p. 73– 89. doi:10.1007/978-1-4939-2556-8_5.
Herbicide tolerance	Canola	-	ODM	2003	Ruiter R, van den Brande I, Stals E, Delauré S, Cornelissen M, D'Halluin K. Spontaneous mutation frequency in plants obscures the effect of chimeraplasty. Plant Mol Biol. 2003;53:675–89. doi:10.1023/b:plan.0000019111.96107.01.
	Cassava	-	CRISPR/Cas9 SDN3	2018	Hummel AW, Chauhan RD, Cermak T, Mutka AM, Vijayaraghavan A, Boyher A, et al. Allele exchange at the EPSPS locus confers glyphosate tolerance in cassava. 1467- 7644. 2018;16:1275–82. doi:10.1111/pbi.12868.

Cotton	-	Meganuclease SDN3	2013	D'Halluin K, Vanderstraeten C, van Hulle J, Rosolowska J, van den Brande I, Pennewaert A, et al. Targeted molecular trait stacking in cotton through targeted doublestrand break induction. Plant Biotechnol J. 2013;11:933–41. doi:10.1111/pbi.12085.
Linum	-	CRISPR/Cas9 SDN1	2016	Sauer NJ, Narváez-Vásquez J, Mozoruk J, Miller RB, Warburg ZJ, Woodward MJ, et al. Oligonucleotide-Mediated Genome Editing Provides Precision and Function to Engineered Nucleases and Antibiotics in Plants. Plant Physiol. 2016;170:1917–28. doi:10.1104/pp.15.01696. 34
Maize	Cause sensitivity	CRISPRnCas9-RT	2020	Jiang, Y.-Y.; Chai, Y.-P.; Lu, M.-H.; Han, X.-L.; Lin, Q.; Zhang, Y.; Zhang, Q.; Zhou, Y.; Wang, X.-C.; Gao, C. Prime editing efficiently generates W542L and S621I double mutations in two ALS genes in maize. Genome Biol. 2020, 21 (1), 1-10.
Maize	Cause sensitivity	CRISPRCasmediated base editing	2019	Li, Y.; Zhu, J.; Wu, H.; Liu, C.; Huang, C.; Lan, J.; Zhao, Y.; Xie, C. Precise base editing of non-allelic acetolactate synthase genes confers sulfonylurea herbicide resistance in maize. Crop J. 2020, 8 (3), 449-456.
Maize	Cause sensitivity	CRISPR-Cas9	2020	Svitashev, S.; Young, J. K.; Schwartz, C.; Gao, H.; Falco, S. C.; Cigan, A. M. Targeted mutagenesis, precise gene editing, and site-specific gene insertion in maize using Cas9 and guide RNA. Plant Physiol. 2015, 169 (2), 931-945.

Maize	-	ZFN SDN3	2013	Ainley WM, Sastry-Dent L, Welter ME, Murray MG, Zeitler B, Amora R, et al. Trait stacking via targeted genome editing. Plant Biotechnol J. 2013;11:1126–34. doi:10.1111/pbi.12107.
Maize	-	SDN1, SDN2, SDN4	2016	Svitashev S, Schwartz C, Lenderts B, Young JK, Mark Cigan A. Genome editing in maize directed by CRISPR-Cas9 ribonucleoprotein complexes. Nat Commun. 2016;7:1– 7. doi:10.1038/ncomms13275
Maize	-	SDN1, SDN2, SDN3	2015	Svitashev S, Young JK, Schwartz C, Gao H, Falco SC, Cigan AM. Targeted Mutagenesis, Precise Gene Editing, and Site-Specific Gene Insertion in Maize Using Cas9 and Guide RNA. Plant Physiol. 2015;169:931–45. doi:10.1104/pp.15.00793.
Maize	-	ODM	2000	Zhu T, Mettenburg K, Peterson DJ, Tagliani L, Baszczynski CL. Engineering herbicide-resistant maize using chimeric RNA/DNA oligonucleotides. Nat Biotechnol. 2000;18:555–8. doi:10.1038/75435.
Maize	-	ODM	1999	Zhu T, Peterson DJ, Tagliani L, St. Clair G, Baszczynski CL, Bowen B. Targeted manipulation of maize genes in vivo using chimeric RNA/DNA oligonucleotides. Proc. Natl. Acad. Sci. 1999;96:8768–73. doi:10.1073/pnas.96.15.8768.

Oilseed rape	Cause sensitivity	CRISPRCasmediated base editing	2018	Wu, J.; Chen, C.; Xian, G.; Liu, D.; Lin, L.; Yin, S.; Sun, Q.; Fang, Y.; Zhang, H.; Wang, Y. Engineering herbicide-resistant oilseed rape by CRISPR/Cas9-mediated cytosine base-editing. Plant Biotechnol. J. 2020, 18 (9), 1857-1859.
Potato	-	CRISPR/Cas9, TALENs SDN2	2016	Butler NM, Baltes NJ, Voytas DF, Douches DS. Geminivirus-Mediated Genome Editing in Potato (Solanum tuberosum L.) Using Sequence-Specific Nucleases. Front Plant Sci. 2016;7:1–13. doi:10.3389/fpls.2016.01045.
Rice	Cause sensitivity	CRISPRnCas9-RT	2020	Butt, H.; Rao, G. S.; Sedeek, K.; Aman, R.; Kamel, R.; Mahfouz, M. Engineering herbicide resistance via prime editing in rice. Plant Biotechnol. J. 2020, 18 (12), 2370-2372.
Rice	Cause sensitivity	Base-editingmediated gene evolution	2020	Kuang, Y.; Li, S.; Ren, B.; Yan, F.; Spetz, C.; Li, X.; Zhou, X.; Zhou, H. Baseediting-mediated artificial evolution of OsALS1 in planta to develop novel herbicide-tolerant rice germplasms. Mol Plant. 2020, 13 (4), 565-572.
Rice	Cause sensitivity	CRISPR-Cpf1	2015	Li, S.; Li, J.; He, Y.; Xu, M.; Zhang, J.; Du, W.; Zhao, Y.; Xia, L. Precise gene replacement in rice by RNA transcript-templated homologous recombination. Nat. Biotechnol. 2019, 37 (4), 445-450.
Rice	Cause sensitivity	CRISPR-Cas9	2019	Sun, Y.; Zhang, X.; Wu, C.; He, Y.; Ma, Y.; Hou, H.; Guo, X.; Du, W.; Zhao, Y.; Xia, L. Engineering herbicide-resistant rice plants through CRISPR/Cas9-mediated homologous recombination of acetolactate synthase. Mol

				Plant. 2016, 9 (4), 628-631.
Rice	Cause sensitivity	CRISPR-Cas9 based cytosine base editing	2016	Zhang, R.; Gao, C. Generating herbicide tolerance in rice by base editing. Sci. China Life Sci. 2020.
Rice	-	CRISPR/Cas9 SDN2	2017	Butt H, Eid A, Ali Z, Atia MAM, Mokhtar MM, Hassan N, et al. Efficient CRISPR/Cas9-Mediated Genome Editing Using a Chimeric Single-Guide RNA Molecule. Front Plant Sci. 2017;8:1441. doi:10.3389/fpls.2017.01441. 35
Rice	-	BE	2018	Li C, Zong Y, Wang YP, Jin S, Zhang DB, Song QN, et al. Expanded base editing in rice and wheat using a Cas9-adenosine deaminase fusion. Genome Biol. 2018;19.
Rice	-	CRISPR/Cas9 SDN2	2016	Li J, Meng X, Zong Y, Chen K, Zhang H, Liu J, et al. Gene replacements and insertions in rice by intron targeting using CRISPR-Cas9. NPLANTS. 2016;2:1–6. doi:10.1038/nplants.2016.139.
Rice	-	ODM	2004	Okuzaki A, Toriyama K. Chimeric RNA/DNA oligonucleotide-directed gene targeting in rice. Plant Cell Rep. 2004;22:509–12. doi:10.1007/s00299-003-0698-2.
Rice	-	BE	2017	Shimatani Z, Kashojiya S, Takayama M, Terada R, Arazoe T, Ishii H, et al. Targeted base editing in rice and tomato using a CRISPR-Cas9 cytidine deaminase fusion. Nat Biotechnol.

				2017;35:441–3. doi:10.1038/nbt.3833.
Rice	-	CRISPR/Cas9 SDN2	2016	Sun Y, Zhang X, Wu C, He Y, Ma Y, Hou H, et al. Engineering Herbicide-Resistant Rice Plants through CRISPR/Cas9-Mediated Homologous Recombination of Acetolactate Synthase. Mol Plant. 2016;9:628–31. doi:10.1016/j.molp.2016.01.001.
Rice	-	TALENs SDN2	2015	Wang M, Liu Y, Zhang C, Liu J, Liu X, Wang L, et al. Gene editing by cotransformation of TALEN and chimeric RNA/DNA oligonucleotides on the rice OsEPSPS gene and the inheritance of mutations. PLoS ONE. 2015;10:e0122755. doi:10.1371/journal.pone.0122755.
Soybean	-	SDN3	2017	Chilcoat D, Liu Z-B, Sander J. Use of CRISPR/Cas9 for Crop Improvement in Maize and Soybean. Prog Mol Biol Transl Sci. 2017;149:27–46. doi:10.1016/bs.pmbts.2017.04.005.
Soybean	-	CRISPR/Cas9 SDN2	2015	Li Z, Liu Z-B, Xing A, Moon BP, Koellhoffer JP, Huang L, et al. Cas9-Guide RNA Directed Genome Editing in Soybean. Plant Physiol. 2015;169:960–70. doi:10.1104/pp.15.00783.
Tomato	Cause sensitivity	CRISPR-Cas9	2018	Danilo, B.; Perrot, L.; Mara, K.; Botton, E.; Nogué, F.; Mazier, M. Efficient and transgene-free gene targeting using Agrobacterium-mediated delivery of the CRISPR/Cas9 system in tomato. Plant Cell Rep. 2019, 38 (4), 459-462.

	Tomato	-	CRISPR/Cas9 SDN1	2019	Danilo B, Perrot L, Mara K, Botton E, Nogue F, Mazier M. Efficient and transgenefree gene targeting using Agrobacterium-mediated delivery of the CRISPR/Cas9 system in tomato. Plant Cell Rep. 2019;38:459–62. doi:10.1007/s00299-019-02373-6.
	Watermelon	-	CRISPR/Cas9 SDN1	2018	Tian SW, Jiang LJ, Cui XX, Zhang J, Guo SG, Li MY, et al. Engineering herbicideresistant watermelon variety through CRISPR/Cas9-mediated base-editing. Plant Cell Rep. 2018;37:1353–6. doi:10.1007/s00299-018-2299-0.
	Potato	-	CRISPR/Cas9 SDN1	2019	Makhotenko AV, Khromov AV, Snigir EA, Makarova SS, Makarov VV, Suprunova TP, et al. Functional Analysis of Coilin in Virus Resistance and Stress Tolerance of Potato Solanum tuberosum using CRISPR-Cas9 Editing. Dokl Biochem Biophys. 2019;484:88–91. doi:10.1134/S1607672919010241.
Salinity tolerance	Rice	Root growth angle	CRISPR-Cas9	2020	Kitomi, Y.; Hanzawa, E.; Kuya, N.; Inoue, H.; Hara, N.; Kawai, S.; Kanno, N.; Endo, M.; Sugimoto, K.; Yamazaki, T. Root angle modifications by the DRO1 homolog improve rice yields in saline paddy fields. Proc. Natl. Acad. Sci. U.S.A. 2020, 117 (35), 21

Rice	Control tolerance	CRISPR-Cas9	2020	Santosh Kumar, V.; Verma, R. K.; Yadav, S. K.; Yadav, P.; Watts, A.; Rao, M.; Chinnusamy, V. CRISPR-Cas9 mediated genome editing of drought and salt tolerance (OsDST) gene in indica mega rice cultivar MTU1010. Physiol. Mol. Biol. Plants 2020, 26, 1099-111
Rice	Cause sensitivity	CRISPR-Cas9	2019	Zhang, A.; Liu, Y.; Wang, F.; Li, T.; Chen, Z.; Kong, D.; Bi, J.; Zhang, F.; Luo, X.; Wang, J. Enhanced rice salinity tolerance via CRISPR/Cas9-targeted mutagenesis of the OsRR22 gene. Mol. Breed. 2019, 39 (3), 1-10.
Rice	-	CRISPR/Cas9 SDN1	2016	Duan Y-B, Li J, Qin R-Y, Xu R-F, Li H, Yang Y-C, et al. Identification of a regulatory element responsible for salt induction of rice OsRAV2 through ex situ and in situ promoter analysis. Plant Mol Biol. 2016;90:49–62. doi:10.1007/s11103-015-0393-z.
Rice	-	CRISPR/Cas9 SDN1	2019	Zhang AN, Liu Y, Wang FM, Li TF, Chen ZH, Kong DY, et al. Enhanced rice salinity tolerance via CRISPR/Cas9-targeted mutagenesis of the OsRR22 gene. Mol Breeding. 2019;39.
Tomato	Cause sensitivity	CRISPR-Cas9	2019	Bouzroud, S.; Gasparini, K.; Hu, G.; Barbosa, M. A. M.; Rosa, B. L.; Fahr, M.; Bendaou, N.; Bouzayen, M.; Zsögön, A.; Smouni, A. Down regulation and loss of auxin response factor 4 function using CRISPR/Cas9 alters plant growth, stomatal function and improves tomato tolerance to salinity and osmotic stress. Genes 2020, 11 (3), 272

Basic Research	-	-	MPK gene family	Cas9- and Cpf1	2018	Ding, D.; Chen, K.; Chen, Y.; Li, H.; Xie, K. Engineering introns to express RNA guides for Cas9-and Cpf1-mediated multiplex genome editing. Mol Plant. 2018, 11 (4), 542552.
	-	African Rice	-	CRISPR/Cas9	2016	Lacchini, E.; Kiegle, E.; Castellani, M.; Adam, H.; Jouannic, S.; Gregis, V.; Kater, M. M. CRISPR-mediated accelerated domestication of African rice landraces. PLoS One 2020, 15 (3), e0229782.
	-	Arabidopsis thaliana	-	CRISPR/Cas9	2021	Beying, N.; Schmidt, C.; Pacher, M.; Houben, A.; Puchta, H. CRISPR–Cas9mediated induction of heritable chromosomal translocations in Arabidopsis. Nat. Plants 2020, 6 (6), 638-645.
	-	Arabidopsis thaliana	-	CRISPR/Cas9	2020	Durr, J.; Papareddy, R.; Nakajima, K.; Gutierrez-Marcos, J. Highly efficient heritable targeted deletions of gene clusters and non-coding regulatory regions in Arabidopsis using CRISPR/Cas9. Sci. Rep. 2018, 8 (1), 1-11.
	-	Arabidopsis thaliana	MPK genes	CRISPR/Cas9	2018	Minkenberg, B.; Xie, K.; Yang, Y. Discovery of rice essential genes by characterizing a CRISPR-edited mutation of closely related rice MAP kinase genes. Plant J. 2017, 89 (3), 636-648.
	-	Arabidopsis thaliana	-	CRISPR/Cas9	2020	Scheben, A.; Hojsgaard, D. Can we use gene-editing to induce apomixis in sexual plants? Genes 2020, 11 (7), 781.
	-	Arabidopsis thaliana	-	CRISPR/Cas9	2020	Schmidt, C.; Pacher, M.; Puchta, H. Efficient induction of heritable inversions in plant genomes using the CRISPR/Cas system. Plant J. 2019, 98 (4), 577-589.

-	Arabidopsis thaliana	-	CRISPR/Cas9	2019	Wu, R.; Lucke, M.; Jang, Y.-t.; Zhu, W.; Symeonidi, E.; Wang, C.; Fitz, J.; Xi, W.; Schwab, R.; Weigel, D. An efficient CRISPR vector toolbox for engineering large deletions in Arabidopsis thaliana. Plant Methods 2018, 14 (1), 1-9.
-	Arabidopsis thaliana	Ribosomal Protein Large 10 subunits	CRISPR/Cas9	2019	Yu, Z.; Chen, Q.; Chen, W.; Zhang, X.; Mei, F.; Zhang, P.; Zhao, M.; Wang, X.; Shi, N.; Jackson, S. Multigene editing via CRISPR/Cas9 guided by a single-sgRNA seed in Arabidopsis. J. Integr. Plant Biol. 2018, 60 (5), 376-381.
-	Arabidopsis thaliana	ABA receptor genes	CRISPR/Cas9	2017	Zhang, Z.; Mao, Y.; Ha, S.; Liu, W.; Botella, J. R.; Zhu, J.-K. A multiplex CRISPR/Cas9 platform for fast and efficient editing of multiple genes in Arabidopsis. Plant Cell Rep. 2016, 35 (7), 1519-1533.
-	Banana	Bacterial Blight	CRISPR/Cas9	2019	Toda, E.; Koiso, N.; Takebayashi, A.; Ichikawa, M.; Kiba, T.; Osakabe, K.; Osakabe, Y.; Sakakibara, H.; Kato, N.; Okamoto, T. An efficient DNA-and selectablemarker-free genome-editing system using zygotes in rice. Nat. Plants 2019, 5 (4), 363-368.
-	Barely	Cytokinin oxidase/dehydrogenase	CRISPR/Cas9	2019	Gasparis, S.; Kała, M.; Przyborowski, M.; Łyżnik, L. A.; Orczyk, W.; NadolskaOrczyk, A. A simple and efficient CRISPR/Cas9 platform for induction of single and multiple, heritable mutations in barley (Hordeum vulgare L.). Plant Methods 2018, 14 (1), 114.

-	Brassica	Resistance to Sclerotinia sclerotiorum	CRISPR/Cas9	2018	Sun, Q.; Lin, L.; Liu, D.; Wu, D.; Fang, Y.; Wu, J.; Wang, Y. CRISPR/Cas9mediated multiplex genome editing of the BnWRKY11 and BnWRKY70 Genes in Brassica napus L. Int. J. Mol. Sci. 2018, 19 (9), 2716.
-	Brassica napus	-	CRISPR/Cas9	2018	Sehgal, N.; Singh, S. Progress on deciphering the molecular aspects of cell-to-cell communication in Brassica self-incompatibility response. 3 Biotech 2018, 8 (8), 1-17. 126. Chen, F.; Yang, Y.; Li, B.; Liu, Z.; Khan, F.; Zhang, T.; Zhou, G.; Tu, J.; She
-	Brassica oleracea	-	CRISPR/Cas9	2018	Ma, C.; Zhu, C.; Zheng, M.; Liu, M.; Zhang, D.; Liu, B.; Li, Q.; Si, J.; Ren, X.; Song, H. CRISPR/Cas9-mediated multiple gene editing in Brassica oleracea var. capitata using the endogenous tRNA-processing system. Hortic. Res. 2019, 6 (1), 1-15.
	Cabbage	-	CRISPR/Cas9	2020	Ma, C.; Liu, M.; Li, Q.; Si, J.; Ren, X.; Song, H. Efficient BoPDS gene editing in cabbage by the CRISPR/Cas9 system. Hortic. Plant J. 2019, 5 (4), 164-169.
-	Catharanthus roseus	Heavy Metals	CRISPR/Cas9	2019	Richter, J.; Watson, J. M.; Stasnik, P.; Borowska, M.; Neuhold, J.; Berger, M.; Stolt-Bergner, P.; Schoft, V.; Hauser, M.-T. Multiplex mutagenesis of four clustered CrRLK1L with CRISPR/Cas9 exposes their growth regulatory roles in response to metal ions. Sci. Rep. 2018, 8 (1), 1-14.

-	Coffea canephora	-	CRISPR/Cas9	2016	Breitler, J.-C.; Dechamp, E.; Campa, C.; Rodrigues, L. A. Z.; Guyot, R.; Marraccini, P.; Etienne, H. CRISPR/Cas9-mediated efficient targeted mutagenesis has the potential to accelerate the domestication of Coffea canephora. PLANT CELL TISS. ORG. 2018, 134 (
-	Durum wheat	Reduce Allergen Proteins	CRISPR/Cas9	2018	Camerlengo, F.; Frittelli, A.; Sparks, C.; Doherty, A.; Martignago, D.; Larré, C.; Lupi, R.; Sestili, F.; Masci, S. CRISPR-Cas9 multiplex editing of the α-amylase/trypsin inhibitor genes to reduce allergen proteins in durum wheat. Front. sustain. food syst. 2020, 4, 104.
-	Hieracium	-	CRISPR/Cas9	2018	Henderson, S. W.; Henderson, S. T.; Goetz, M.; Koltunow, A. M. Efficient CRISPR/Cas9-mediated knockout of an endogenous PHYTOENE DESATURASE gene in T1 progeny of apomictic Hieracium enables new strategies for apomixis gene identification. Genes 2020, 11 (9), 1
-	Kiwi Fruit	Phytoene desaturase	CRISPR/Cas9	2019	Wang, Z.; Wang, S.; Li, D.; Zhang, Q.; Li, L.; Zhong, C.; Liu, Y.; Huang, H. Optimized paired-sgRNA/Cas9 cloning and expression cassette triggers high-efficiency multiplex genome editing in kiwifruit. Plant Biotechnol. J. 2018, 16 (8), 1424-1433.

-	Maize	Temperature sensitivity	LbCas12a	2019	Malzahn, A. A.; Tang, X.; Lee, K.; Ren, Q.; Sretenovic, S.; Zhang, Y.; Chen, H.; Kang, M.; Bao, Y.; Zheng, X. Application of CRISPR-Cas12a temperature sensitivity for improved genome editing in rice, maize, and Arabidopsis. BMC Biol. 2019, 17 (1), 1-14.
-	Maize	Endosperm-specific core transcription factor	CRISPR/Cas9	2017	Qi, W.; Zhu, T.; Tian, Z.; Li, C.; Zhang, W.; Song, R. High-efficiency CRISPR/Cas9 multiplex gene editing using the glycine tRNA-processing system-based strategy in maize. BMC Biotechnol. 2016, 16 (1), 1-8.
-	Maize	Epicuticle Wax	Cas9 and Cpf1	2020	Toda, E.; Koiso, N.; Takebayashi, A.; Ichikawa, M.; Kiba, T.; Osakabe, K.; Osakabe, Y.; Sakakibara, H.; Kato, N.; Okamoto, T. An efficient DNA-and selectablemarker-free genome-editing system using zygotes in rice. Nat. Plants 2019, 5 (4), 363-368.
-	Maize	-	CRISPR/Cas9	2016	Svitashev, S.; Young, J. K.; Schwartz, C.; Gao, H.; Falco, S. C.; Cigan, A. M. Targeted mutagenesis, precise gene editing, and site-specific gene insertion in maize using Cas9 and guide RNA. Plant Physiol. 2015, 169 (2), 931-945.
-	Medicago sativa	Stay Green Trait	CRISPR/Cas9	2019	Toda, E.; Koiso, N.; Takebayashi, A.; Ichikawa, M.; Kiba, T.; Osakabe, K.; Osakabe, Y.; Sakakibara, H.; Kato, N.; Okamoto, T. An efficient DNA-and selectablemarker-free genome-editing system using zygotes in rice. Nat. Plants 2019, 5 (4), 363-368.

-	N. benthamiana	-	CRISPR/Cas9	2018	Toda, E.; Koiso, N.; Takebayashi, A.; Ichikawa, M.; Kiba, T.; Osakabe, K.; Osakabe, Y.; Sakakibara, H.; Kato, N.; Okamoto, T. An efficient DNA-and selectablemarker-free genome-editing system using zygotes in rice. Nat. Plants 2019, 5 (4), 363-368.
-	Nicotiana benthamiana	NbAGO1 paralogs	CRISPR/Cas9	2020	Cody, W. B.; Scholthof, H. B.; Mirkov, T. E. Multiplexed gene editing and protein overexpression using a tobacco mosaic virus viral vector. Plant Physiol. 2017, 175 (1), 23-35.
-	Nicotiana tabacum	-	CRISPR/Cas9	2019	Ordon, J.; Gantner, J.; Kemna, J.; Schwalgun, L.; Reschke, M.; Streubel, J.; Boch, J.; Stuttmann, J. Generation of chromosomal deletions in dicotyledonous plants employing a user-friendly genome editing toolkit. Plant J. 2017, 89 (1), 155-168.
-	Oilseed Rape	-	CRISPR/Cas9	2019	Sehgal, N.; Singh, S. Progress on deciphering the molecular aspects of cell-to-cell communication in Brassica self-incompatibility response. 3 Biotech 2018, 8 (8), 1-17. 126. Chen, F.; Yang, Y.; Li, B.; Liu, Z.; Khan, F.; Zhang, T.; Zhou, G.; Tu, J.; She
-	Physalis pruinosa	-	CRISPR/Cas9	2020	Lemmon, Z. H.; Reem, N. T.; Dalrymple, J.; Soyk, S.; Swartwood, K. E.; Rodriguez-Leal, D.; Van Eck, J.; Lippman, Z. B. Rapid improvement of domestication traits in an orphan crop by genome editing. Nat. Plants 2018, 4 (10), 766-770.

-	Poa pratensis	-	CRISPR/Cas9	2018	Marconi, G.; Aiello, D.; Kindiger, B.; Storchi, L.; Marrone, A.; Reale, L.; Terzaroli, N.; Albertini, E. The Role of APOSTART in Switching between Sexuality and Apomixis in Poa pratensis. Genes 2020, 11 (8), 941.
-	Potato	-	CRISPR/Cas9	2019	Enciso-Rodriguez, F.; Manrique-Carpintero, N. C.; Nadakuduti, S. S.; Buell, C. R.; Zarka, D.; Douches, D. Overcoming self-incompatibility in diploid potato using CRISPRCas9. Front. Plant Sci. 2019, 10, 376.
-	Potato	Alpha-solanine-free hairy roots	CRISPR/Cas9	2019	Nakayasu, M.; Akiyama, R.; Lee, H. J.; Osakabe, K.; Osakabe, Y.; Watanabe, B.; Sugimoto, Y.; Umemoto, N.; Saito, K.; Muranaka, T. Generation of α-solanine-free hairy roots of potato by CRISPR/Cas9 mediated genome editing of the St16DOX gene. Plant Physiol. Biochem. 2018, 131, 70-77
-	Rice		CRISPR/ Cas9	2019	https://www.nature.com/articles/s41477-019-0386-z
-	Rice	-	CRISPR/Cas9	2020	Barman, H. N.; Sheng, Z.; Fiaz, S.; Zhong, M.; Wu, Y.; Cai, Y.; Wang, W.; Jiao, G.; Tang, S.; Wei, X. Generation of a new thermo-sensitive genic male sterile rice line by targeted mutagenesis of TMS5 gene through CRISPR/Cas9 system. BMC Plant Biol. 2019,

-	Rice	-	CRISPR/Cas9	2020	Basnet, R.; Hussain, N.; Shu, Q. OsDGD2β is the sole digalactosyldiacylglycerol synthase gene highly expressed in anther, and its mutation confers male sterility in rice. Rice 2019, 12 (1), 1-13.
-	Rice	-	Cpf1	2016	Begemann, M. B.; Gray, B. N.; January, E.; Gordon, G. C.; He, Y.; Liu, H.; Wu, X.; Brutnell, T. P.; Mockler, T. C.; Oufattole, M. Precise insertion and guided editing of higher plant genomes using Cpf1 CRISPR nucleases. Sci. Rep. 2017, 7 (1), 1-6.
-	Rice	Serine/Arginine gene family	CRISPR/Cas9	2019	Butt, H.; Piatek, A.; Li, L.; SN Reddy, A.; M Mahfouz, M. Multiplex CRISPR mutagenesis of the serine/arginine-rich (SR) gene family in rice. Genes 2019, 10 (8), 596.
-	Rice	Anthocyanin pathway	CRISPR/Cas9	2017	Ma, X.; Zhang, Q.; Zhu, Q.; Liu, W.; Chen, Y.; Qiu, R.; Wang, B.; Yang, Z.; Li, H.; Lin, Y. A robust CRISPR/Cas9 system for convenient, high-efficiency multiplex genome editing in monocot and dicot plants. Mol Plant. 2015, 8 (8), 1274-1284.
-	Rice	-	CRISPR/Cas9	2018	Scheben, A.; Hojsgaard, D. Can we use gene-editing to induce apomixis in sexual plants? Genes 2020, 11 (7), 781.
-	Rice	Herbicide Tolerance	Cas9	2018	Shimatani, Z.; Fujikura, U.; Ishii, H.; Terada, R.; Nishida, K.; Kondo, A. Herbicide tolerance-assisted multiplex targeted nucleotide substitution in rice. Data Brief 2018, 20, 1325-1331.

-	Rice	-	CRISPR/Cas9	2020	Suketomo, C.; Kazama, T.; Toriyama, K. Fertility restoration of Chinese wild ricetype cytoplasmic male sterility by CRISPR/Cas9-mediated genome editing of nuclearencoded RETROGRADE-REGULATED MALE STERILITY. Plant Biotechnol. J. 2020, 20.0326 b.
-	Rice	-	CRISPR/Cas9	2016	Sun, Y.; Zhang, X.; Wu, C.; He, Y.; Ma, Y.; Hou, H.; Guo, X.; Du, W.; Zhao, Y.; Xia, L. Engineering herbicide-resistant rice plants through CRISPR/Cas9-mediated homologous recombination of acetolactate synthase. Mol Plant. 2016, 9 (4), 628-631.
-	Rice	Agronomic traits	CRISPR/Cas9	2020	Toda, E.; Koiso, N.; Takebayashi, A.; Ichikawa, M.; Kiba, T.; Osakabe, K.; Osakabe, Y.; Sakakibara, H.; Kato, N.; Okamoto, T. An efficient DNA-and selectablemarker-free genome-editing system using zygotes in rice. Nat. Plants 2019, 5 (4), 363-368.
-	Rice	Reduction of ingredients harmful to health (arsenic content)	CRISPR/Cas9 SDN1	2017	Wang F-Z, Chen M-X, Yu L-J, Xie L-J, Yuan L-B, Qi H, et al. OsARM1, an R2R3 MYB Transcription Factor, Is Involved in Regulation of the Response to Arsenic Stress in Rice. Front Plant Sci. 2017;8:1868. doi:10.3389/fpls.2017.01868.
-	Rice	-	CRISPR/Cas9	2020	Wang, K. Fixation of hybrid vigor in rice: synthetic apomixis generated by genome editing. aBIOTECH 2020, 1 (1), 15-20.

-	Rice	-	CRISPR/Cas9	2019	Wang, K. Fixation of hybrid vigor in rice: synthetic apomixis generated by genome editing. aBIOTECH 2020, 1 (1), 15-20.
-	Rice	LEA gene family	Cpf1 and Cas9	2018	Wang, M.; Mao, Y.; Lu, Y.; Wang, Z.; Tao, X.; Zhu, J. K. Multiplex gene editing in rice with simplified CRISPR-Cpf1 and CRISPR-Cas9 systems. J. Integr. Plant Biol. 2018, 60 (8), 626-631.
-	Rice	Cellular Signalling Pathways	CRISPR/Cas9	2017	Xie, K.; Minkenberg, B.; Yang, Y. Boosting CRISPR/Cas9 multiplex editing capability with the endogenous tRNA-processing system. Proc. Natl. Acad. Sci. U.S.A. 2015, 112 (11), 3570-3575.
-	Rice	-	CRISPR/Cas9	2020	Xuan, C.; Liu, Y.; Liu, C.; Lusuwi, L. B. T.; Fu, X.; Chen, X.; Ma, B. Identification and gene cloning of a male-sterile mutant Oswbc11 in rice. Plant Breed. 2019, 138 (3), 290-298.
-	Rice	Agronomic Parameters	CRISPR/Cas9	2019	Zhou, J.; Xin, X.; He, Y.; Chen, H.; Li, Q.; Tang, X.; Zhong, Z.; Deng, K.; Zheng, X.; Akher, S. A. Multiplex QTL editing of grain-related genes improves yield in elite rice varieties. Plant Cell Rep. 2019, 38 (4), 475-485.
-	Rice	-	CRISPR/Cas9	2015	Dong, O. X.; Yu, S.; Jain, R.; Zhang, N.; Duong, P. Q.; Butler, C.; Li, Y.; Lipzen, A.; Martin, J. A.; Barry, K. W. Marker-free carotenoid-enriched rice generated through targeted gene insertion using CRISPR-Cas9. Nat. Commun. 2020, 11 (1), 1-10.

-	Rice	-	CRISPR/Cas9	2015	Li, J.; Meng, X.; Zong, Y.; Chen, K.; Zhang, H.; Liu, J.; Li, J.; Gao, C. Gene replacements and insertions in rice by intron targeting using CRISPR–Cas9. Nat. Plants 2016, 2 (10), 1-6.
-	Rice	Thermogenic male sterility	CRISPR/Cas9	2019	Li, S.; Shen, L.; Hu, P.; Liu, Q.; Zhu, X.; Qian, Q.; Wang, K.; Wang, Y. Developing disease-resistant thermosensitive male sterile rice by multiplex gene editing. J. Integr. Plant Biol. 2019, 61 (12), 1201-1205.
-	Rice	Protein kinases	CRISPR/Cas9	2019	Toda, E.; Koiso, N.; Takebayashi, A.; Ichikawa, M.; Kiba, T.; Osakabe, K.; Osakabe, Y.; Sakakibara, H.; Kato, N.; Okamoto, T. An efficient DNA-and selectablemarker-free genome-editing system using zygotes in rice. Nat. Plants 2019, 5 (4), 363-368.
	Rice O. sativa & O. japonica crossed with O. glaberrima	-	CRISPR/Cas9	2020	Xie, Y.; Xu, P.; Huang, J.; Ma, S.; Xie, X.; Tao, D.; Chen, L.; Liu, Y.-G. Interspecific hybrid sterility in rice is mediated by OgTPR1 at the S1 locus encoding a peptidase-like protein. Mol Plant. 2017, 10 (8), 1137-1140.
-	Rice O. sativa and O. japonica cross	-	CRISPR/Cas9	2019	Shen, R.; Wang, L.; Liu, X.; Wu, J.; Jin, W.; Zhao, X.; Xie, X.; Zhu, Q.; Tang, H.; Li, Q. Genomic structural variation-mediated allelic suppression causes hybrid male sterility in rice. Nat. Commun. 2017, 8 (1), 1-10.

-	Rye Grass	-	CRISPR/Cas9	2018	Zhang, Y.; Ran, Y.; Nagy, I.; Lenk, I.; Qiu, J. L.; Asp, T.; Jensen, C. S.; Gao, C. Targeted mutagenesis in ryegrass (Lolium spp.) using the CRISPR/Cas9 system. Plant Biotechnol. J. 2020, 18 (9), 1854-1856.
-	S. pimpinellifolium	-	CRISPR/Cas9	2015	Li, T.; Yang, X.; Yu, Y.; Si, X.; Zhai, X.; Zhang, H.; Dong, W.; Gao, C.; Xu, C. Domestication of wild tomato is accelerated by genome editing. Nat. Biotechnol. 2018, 36 (12), 1160-1163.
-	S. pimpinellifolium	-	CRISPR/Cas9	2017	Zsögön, A.; Čermák, T.; Naves, E. R.; Notini, M. M.; Edel, K. H.; Weinl, S.; Freschi, L.; Voytas, D. F.; Kudla, J.; Peres, L. E. P. De novo domestication of wild tomato using genome editing. Nat. Biotechnol. 2018, 36 (12), 1211-1216.
-	Soybean	Flowering time	CRISPR/Cas9	2018	Toda, E.; Koiso, N.; Takebayashi, A.; Ichikawa, M.; Kiba, T.; Osakabe, K.; Osakabe, Y.; Sakakibara, H.; Kato, N.; Okamoto, T. An efficient DNA-and selectablemarker-free genome-editing system using zygotes in rice. Nat. Plants 2019, 5 (4), 363-368.
-	Soybean	Soybean mosaic virus	CRISPR/Cas9	2019	Zhang, P.; Du, H.; Wang, J.; Pu, Y.; Yang, C.; Yan, R.; Yang, H.; Cheng, H.; Yu, D. Multiplex CRISPR/Cas9-mediated metabolic engineering increases soya bean isoflavone content and resistance to soya bean mosaic virus. Plant Biotechnol. J. 2020, 18 (6),

-	Tobacco	Homozygous multiplex genome editing in polyploid	Cas9 and Cpf1	2019	Toda, E.; Koiso, N.; Takebayashi, A.; Ichikawa, M.; Kiba, T.; Osakabe, K.; Osakabe, Y.; Sakakibara, H.; Kato, N.; Okamoto, T. An efficient DNA-and selectablemarker-free genome-editing system using zygotes in rice. Nat. Plants 2019, 5 (4), 363-368.
-	Tomato	Lycopene synthesis	CRISPR/Cas9	2018	Li, X.; Wang, Y.; Chen, S.; Tian, H.; Fu, D.; Zhu, B.; Luo, Y.; Zhu, H. Lycopene is enriched in tomato fruit by CRISPR/Cas9-mediated multiplex genome editing. Front. Plant Sci. 2018, 9, 559.
-	Tomato	-	CRISPR/Cas9	2018	Qin, X.; Li, W.; Liu, Y.; Tan, M.; Ganal, M.; Chetelat, R. T. A farnesyl pyrophosphate synthase gene expressed in pollen functions in S-RN ase-independent unilateral incompatibility. Plant J. 2018, 93 (3), 417-430.
-	Tomato	-	CRISPR/Cas9	2015	Čermák, T.; Baltes, N. J.; Čegan, R.; Zhang, Y.; Voytas, D. F. High-frequency, precise modification of the tomato genome. Genome Biol. 2015, 16 (1), 1-15.
-	Tomato	γ-aminobutyric acid	CRISPR/Cas9	2018	Li, R.; Li, R.; Li, X.; Fu, D.; Zhu, B.; Tian, H.; Luo, Y.; Zhu, H. Multiplexed CRISPR/Cas9-mediated metabolic engineering of γ-aminobutyric acid levels in Solanum lycopersicum. Plant Biotechnol. J. 2018, 16 (2), 415-427.

-	Tomato	-	CRISPR/Cas9	2017	Ueta, R.; Abe, C.; Watanabe, T.; Sugano, S. S.; Ishihara, R.; Ezura, H.; Osakabe, Y.; Osakabe, K. Rapid breeding of parthenocarpic tomato plants using CRISPR/Cas9. Sci. Rep. 2017, 7 (1), 1-8.
-	Wheat	Growth and development related multiple traits	CRISPR/Cas9	2018	Li, J.; Zhang, S.; Zhang, R.; Gao, J.; Qi, Y.; Song, G.; Li, W.; Li, Y.; Li, G. Efficient multiplex genome editing by CRISPR/Cas9 in common wheat. Plant Biotechnol. J. 2020.
-	Wheat	Multiple traits	CRISPR/Cas9	2017	Wang, W.; Akhunova, A.; Chao, S.; Akhunov, E. Optimizing multiplex CRISPR/Cas9-based genome editing for wheat. BioRxiv 2016, 051342.
-	Wheat	Agronomic traits	CRISPR/Cas9	2018	Wang, W.; Pan, Q.; He, F.; Akhunova, A.; Chao, S.; Trick, H.; Akhunov, E. Transgenerational CRISPR-Cas9 activity facilitates multiplex gene editing in allopolyploid wheat. CRISPR J. 2018, 1 (1), 65-74.
-	Zea mays	-	CRISPR/Cas9	2019	Schwartz, C.; Lenderts, B.; Feigenbutz, L.; Barone, P.; Llaca, V.; Fengler, K.; Svitashev, S. CRISPR–Cas9-mediated 75.5-Mb inversion in maize. Nat. Plants 2020, 6 (12), 1427-1431.
