# Supplementary material for: Genome editing techniques in plants: a comprehensive review and future prospects toward zero hunger
Source: GM Crops Food. 2022 Feb 9;12(2):601–15. doi: 10.1080/21645698.2021.2021724 (PMC9208631; doi:10.1080/21645698.2021.2021724)
Supplement: Supplemental Material [file KGMC_A_2021724_SM8963.zip › Table_S2.pdf]

Table S2: Summary of role of CRISPR system in biotic stress improvementt

Trait	Plant	Gene Function	Technique	Year	References
	Rice	Disease facilitation	CRISPR-Cas9	2018	Li, C.; Li, W.; Zhou, Z.; Chen, H.; Xie, C.; Lin, Y. A new rice breeding method: CRISPR/Cas9 system editing of the Xa13 promoter to cultivate transgene-free bacterial blight-resistant rice. Plant Biotechnol. J. 2020, 18 (2), 313.
Bacterial blight	Rice	Disease facilitation	CRISPR-Cas9	2018	Zeng, X.; Luo, Y.; Vu, N. T. Q.; Shen, S.; Xia, K.; Zhang, M. CRISPR/Cas9mediated mutation of OsSWEET14 in rice cv. Zhonghua11 confers resistance to Xanthomonas oryzae pv. oryzae without yield penalty. BMC Plant Biol. 2020, 20 (1), 1-11.<br>Zafar, K.; Khan, M. Z.; Amin, I.; Mukhtar, Z.; Yasmin, S.; Arif, M.; Ejaz, K.; Mansoor, S. Precise CRISPR-Cas9 mediated genome editing in super basmati rice for resistance against bacterial blight by targeting the major susceptibility gene. Front. Plant Sci. 2020, 11, 575.
	Rice	Disease facilitation	CRISPR-Cas9	2018	Zhou, J.; Peng, Z.; Long, J.; Sosso, D.; Liu, B.; Eom, J. S.; Huang, S.; Liu, S.; Vera Cruz, C.; Frommer, W. B. Gene targeting by the TAL effector PthXo2 reveals cryptic resistance gene for bacterial blight of rice. Plant J. 2015, 82 (4), 632-643.
	Tomato	Disease facilitation	CRISPR-Cas9	2017	de Toledo Thomazella, D. P.; Brail, Q.; Dahlbeck, D.; Staskawicz, B. CRISPR-Cas9 mediated mutagenesis of a DMR6 ortholog in tomato confers broad-spectrum disease resistance. BioRxiv 2016, 064824.
Bacterial speck					
	Tomato	Disease facilitation	CRISPR-Cas9	2017	Ortigosa, A.; Gimenez-Ibanez, S.; Leonhardt, N.; Solano, R. Design of a bacterial speck resistant tomato by CRISPR/Cas9-mediated editing of Sl JAZ 2. Plant Biotechnol. J. 2019, 17 (3), 665-673.

	Banana	Banana streak virus	CRISPR/Cas9	2016	Tripathi, L., Ntui, V. O., and Tripathi, J. N. (2019). Application of genetic modification and genome editing for developing climate-smart banana. Food Energy Secur. 8:e00168. doi: 10.1002/fes3.168
	banana		CRISPR/Cas9	2020	https://www.sciencedirect.com/science/article/abs/pii/S1369526620300558
	Banana	Resistance against Banana Streak Virus (BSV)	CRISPR/Cas9 SDN1	2019	Tripathi JN, Ntui VO, Ron M, Muiruri SK, Britt A, Tripathi L. CRISPR/Cas9 editing of endogenous banana streak virus in the B genome of Musa spp. overcomes a major challenge in banana breeding 2019. doi:10.1038/s42003-019-0288-7.
Disease resistance	Barley	Broad spectrum	CRISPR/Cas9	2020	Kumar, N., Galli, M., Ordon, J., Stuttmann, J., Kogel, K., and Imani, J. (2018). Further analysis of barley MORC1 using a highly efficient RNA-guided Cas9 Gene-editing system. Plant Biotechnol. J. 16, 1892–1903. doi: 10.1111/pbi.12924
	Barley	Virus spread	CRISPR-Cas9	2017	Kis, A.; Hamar, É.; Tholt, G.; Bán, R.; Havelda, Z. Creating highly efficient resistance against wheat dwarf virus in barley by employing CRISPR/Cas9 system. Plant Biotechnol. J. 2019, 17 (6), 1004.
	Barley	Resistance against Wheat Dwarf Virus	CRISPR/Cas9 SDN1	2019	Kis A, Hamar E, Tholt G, Ban R, Havelda Z. Creating highly efficient resistance against wheat dwarf virus in barley by employing CRISPR/Cas9 system. 1467-7644. 2019;17:1004–6. doi:10.1111/pbi.13077.
	Cacao	Resistance to phytopthora	CRISPR/Cas9	2016	Fister, A. S., Landherr, L., Maximova, S. N., and Guiltinan, M. J. (2018). Transient expression of CRISPR/Cas9 machinery targeting TcNPR3 enhances defense response in theobroma cacao. Front. Plant Sci. 9:268. doi: 10.3389/fpls.2018.00268

cacao	Phytophthora tropicalis resistance	CRISPR/Cas9 SDN1	2018	Fister AS, Landherr L, Maximova SN, Guiltinan MJ. Transient Expression of CRISPR/Cas9 Machinery Targeting TcNPR3 Enhances Defense Response in Theobroma cacao. Front. Plant Sci. 2018;9:47. doi:10.3389/fpls.2018.00268.
Canola	Sclerotinia sclerotiorium resistance	CRISPR/Cas9 SDN1	2018	Sun QF, Lin L, Liu DX, Wu DW, Fang YJ, Wu J, Wang YP. CRISPR/Cas9-Mediated Multiplex Genome Editing of the BnWRKY11 and BnWRKY70 Genes in Brassica napus L. Int J Mol Sci. 2018;19.
Cassava	Cassava brown streak disease	CRISPR/Cas9	2019	Gomez, M. A., Lin, Z. D., Moll, T., Chauhan, R. D., Hayden, L., Renninger, K., et al. (2019). Simultaneous CRISPR/Cas9-mediated editing of cassava EIF4E isoforms NCBP-1 and NCBP-2 reduces cassava brown streak disease symptom severity and incidence. Plant Biotechnol. J. 17, 421–434. doi: 10.1111/pbi.12987
Cotton	Verticillium dahliae	CRISPR/Cas9	2013	Zhang, Z., Ge, X., Luo, X., Wang, P., Fan, Q., Hu, G., et al. (2018). Simultaneous editing of two copies of Gh14-3-3d confers enhanced transgene-clean plant defense against verticillium dahliae in allotetraploid upland cotton. Front. Plant Sci. 9:842. doi: 10.3389/fpls.2018.00842
Cotton	Resistance against Verticillium dahlia	CRISPR/Cas9 SDN1	2018	Zhang ZN, Ge XY, Luo XL, Wang P, Fan Q, Hu G, et al. Simultaneous Editing of Two Copies of Gh14-3-3d Confers Enhanced Transgene-Clean Plant Defense Against Verticillium dahliae in Allotetraploid Upland Cotton. Front Plant Sci. 2018;9.
Cucumber	Broad spectrum viral resistance	CRISPR/Cas9	2016	Chandrasekaran, J., Brumin, M., Wolf, D., Leibman, D., Klap, C., Pearlsman, M., et al. (2016). Development of broad virus resistance in non-transgenic cucumber using CRISPR/Cas9 technology. Mol. Plant Pathol. 17, 1140–1153. doi: 10.1111/mpp.12375

Grape	Powdery mildew	CRISPR/Cas9	2017	Wan, D.-Y., Guo, Y., Cheng, Y., Hu, Y., Xiao, S., Wang, Y., et al. (2020). CRISPR/Cas9-mediated mutagenesis of VvMLO3 results in enhanced resistance to powdery mildew in grapevine (Vitis Vinifera). Hortic. Res. 7, 1–14. doi: 10.1038/s41438-020-0339-8
Grapefruit	Citrus cancer resistance	CRISPR/Cas9 SDN1	2016	Jia H, Orbovic V, Jones JB, Wang N. Modification of the PthA4 effector binding elements in Type I CsLOB1 promoter using Cas9/sgRNA to produce transgenic Duncan 32 grapefruit alleviating XccΔpthA4:dCsLOB1.3 infection. Plant Biotechnol J. 2016;14:1291–301. doi:10.1111/pbi.12495.
Grapefruit	Citrus cancer resistance	CRISPR/Cpf1 SDN1	2019	Jia H, Orbovic V, Wang N. CRISPR-LbCas12a-mediated modification of citrus. 1467- 7644 2019. doi:10.1111/pbi.13109.
Grapefruit	Citrus cancer resistance	CRISPR/Cas9 SDN2	2017	Jia H, Zhang Y, Orbović V, Xu J, White FF, Jones JB, Wang N. Genome editing of the disease susceptibility gene CsLOB1 in citrus confers resistance to citrus canker. Plant Biotechnol J. 2017;15:817–23. doi:10.1111/pbi.12677.
Grapevine	Gray mold resistance	CRISPR/Cas9 SDN1	2018	Wang X, Tu M, Wang D, Liu J, Li Y, Li Z, et al. CRISPR/Cas9-mediated efficient targeted mutagenesis in grape in the first generation. Plant Biotechnol J. 2018;16:844–55. doi:10.1111/pbi.12832.
Orange	Citrus cancer resistance	CRISPR/Cas9 SDN1	2017	Peng A, Chen S, Lei T, Xu L, He Y, Wu L, et al. Engineering canker-resistant plants through CRISPR/Cas9-targeted editing of the susceptibility gene CsLOB1 promoter in citrus. Plant Biotechnol J. 2017;15:1509–19. doi:10.1111/pbi.12733.

Potato	Potato virus Y	CRISPR/Cas9	2018	Makhotenko, A. V., Khromov, A. V., Snigir, E. A., Makarova, S. S., Makarov, V. V., Suprunova, T. P., et al. (2019). Functional analysis of coilin in virus resistance and stress tolerance of potato solanum tuberosum using CRISPR-Cas9 editing. Dokl. Biochem. Biophys. 484, 88–91. doi: 10.1134/S1607672919010241
Rice	Bacterial blight resistance	CRISPR/Cas9	2019	Jiang, W., Zhou, H., Bi, H., Fromm, M., Yang, B., and Weeks, D. P. (2013). Demonstration of CRISPR/Cas9/SgRNA-mediated targeted gene modification in arabidopsis, tobacco, sorghum and rice. Nucleic Acids Res. 41:e188. doi: 10.1093/nar/gkt780
Rice	Bacterial blight	CRISPR/Cas9	2014	Kim, Y.-A., Moon, H., and Park, C.-J. (2019). CRISPR/Cas9-targetec mutagenesis of Os8N3 in rice to confer resistance to xanthomonas oryzae pv. Oryzae. Rice 12:67. doi: 10.1186/s12284-019-0325-7
Rice	Broad spectrum	CRISPR/Cas9	2016	Kumar, N., Galli, M., Ordon, J., Stuttmann, J., Kogel, K., and Imani, J. (2018). Further analysis of barley MORC1 using a highly efficient RNA-guided Cas9 Gene-editing system. Plant Biotechnol. J. 16, 1892–1903. doi: 10.1111/pbi.12924<br><br>Zhou, X., Liao, H., Chern, M., Yin, J., Chen, Y., Wang, J., et al. (2018). Loss of function of a rice TPR-Domain RNA-binding protein confers broadspectrum disease resistance. Proc. Natl. Acad. Sci. U.S.A. 115, 3174–3179. doi: 10.1073/pnas.1705927115
Rice	Rice tungro spherical virus	CRISPR/Cas9	2017	Macovei, A., Sevilla, N. R., Cantos, C., Jonson, G. B., Slamet-Loedin, I., Cermák, T., et al. (2018). Novel alleles of rice EIF4G generated by CRISPR/Cas9-targeted mutagenesis confer resistance to rice tungro spherical virus. Plant Biotechnol. J. 16, 1918–1927. doi: 10.1111/pbi.12927

Rice	Bacterial blight	CRISPR/Cas9	2019	Oliva, R., Ji, C., Atienza-Grande, G., Huguet-Tapia, J. C., Perez-Quintero, A., Li, T., et al. (2019). Broad-spectrum resistance to bacterial blight in rice using genome editing. Nat. Biotechnol. 37, 1344–1350. doi: 10.1038/s41587-019-0267-z
Rice	Rice leaf blast resistance	CRISPR/Cas9	2019	Wang, F., Wang, C., Liu, P., Lei, C., Hao, W., Gao, Y., et al. (2016). Enhanced rice blast resistance by CRISPR/Cas9-targeted mutagenesis of the ERF transcription factor gene OsERF922. PLOS ONE 11:e0154027. doi: 10.1371/journal.pone.0154027
Rice	Bacterial blight	TALENs	2018	Xu, Z., Xu, X., Gong, Q., Li, Z., Li, Y., Wang, S., et al. (2019). Engineering broadspectrum bacterial blight resistance by simultaneously disrupting variable TALE-binding elements of multiple susceptibility genes in rice. Mol. Plant 12, 1434–1446. doi: 10.1016/j.molp.2019.08.006
Rice	Facilitate pathogen growth	CRISPR-Cas9	2016	Liang, Y.; Han, Y.; Wang, C.; Jiang, C.; Xu, J.-R. Targeted deletion of the USTA and UvSLT2 genes efficiently in Ustilaginoidea virens with the CRISPR-Cas9 system. Front. Plant Sci. 2018, 9, 699.
Rice	Resistance to rice browning	CRISPR/Cas9; SDN1	2016	Wang F, Wang C, Liu P, Lei C, Hao W, GAO Y, et al. Enhanced Rice Blast Resistance by CRISPR/Cas9-Targeted Mutagenesis of the ERF Transcription Factor Gene OsERF922. PLoS ONE. 2016;11:e0154027. doi:10.1371/journal.pone.0154027.
Rice	Resistance against bacterial blight	TALENs SDN1	2017	Blanvillain-Baufumé S, Reschke M, Solé M, Auguy F, Doucoure H, Szurek B, et al. Targeted promoter editing for rice resistance to Xanthomonas oryzae pv. oryzae reveals differential activities for SWEET14-inducing TAL effectors. Plant Biotechnol J. 2017;15:306–17. doi:10.1111/pbi.12613.

Rice	Xanthomonas RS105 resistance	TALENs SDN1	2017	Cai L, Cao Y, Xu Z, Ma W, Zakria M, Zou L, et al. A Transcription Activator-Like Effector Tal7 of Xanthomonas oryzae pv. oryzicola Activates Rice Gene Os09g29100 to Suppress Rice Immunity. Sci Rep. 2017;7:5089. doi:10.1038/s41598-017-04800-8.
Rice	Resistance against bacterial blight	CRISPR/Cas9 SDN1	2018	iao Y, Bai Q, Xu P, Wu T, Guo D, Peng Y, et al. Mutation in Rice Abscisic Acid2 Results in Cell Death, Enhanced Disease-Resistance, Altered Seed Dormancy and Development. Front. Plant Sci. 2018;9:1248. doi:10.3389/fpls.2018.00405.
Rice	Resistance against bacterial blight	TALENs SDN1	2019	Li S, Shen L, Hu P, Liu Q, Zhu X, Qian Q, et al. Developing disease-resistant thermosensitive male sterile rice by multiplex gene editing 2019. doi:10.1111/jipb.12774.
Rice	Resistance against bacterial blight	TALENs SDN1	2012	Li T, Liu B, Spalding MH, Weeks DP, Yang B. High-efficiency TALEN-based gene editing produces disease-resistant rice. Nat Biotechnol. 2012;30:390–2. doi:10.1038/nbt.2199. 33
Rice	Resistance against bacterial blight	TALENs SDN1	2017	Wang J, Tian D, Gu K, Yang X, Wang L, Zeng X, Yin Z. Induction of Xa10-like Genes in Rice Cultivar Nipponbare Confers Disease Resistance to Rice Bacterial Blight. Mol Plant Microbe Interact. 2017;30:466–77. doi:10.1094/MPMI-11-16-0229-R.
Rice	Resistance against bacterial blight	TALENs SDN2	2017	Xie C, Zhang G, Zhang Y, Song X, Guo H, Chen X, Fang R. SRWD1, a novel target gene of DELLA and WRKY proteins, participates in the development and immune response of rice (Oryza sativa L.). Science Bulletin. 2017;62:1639–48. doi:10.1016/j.scib.2017.12.002.
Rice	Resistance against bacterial blight	CRISPR/Cas9; SDN1	2015	Zhou J, Peng Z, Long J, Sosso D, Liu B, Eom J-S, et al. Gene targeting by the TAL effector PthXo2 reveals cryptic resistance gene for bacterial blight of rice. Plant J. 2015;82:632–43. doi:10.1111/tpj.12838.

Rice	Disease facilitation	CRISPR-Cas9	2019	Macovei, A.; Sevilla, N. R.; Cantos, C.; Jonson, G. B.; Slamet-Loedin, I.; Čermák, T.; Voytas, D. F.; Choi, I. R.; Chadha-Mohanty, P. Novel alleles of rice eIF4G generated by CRISPR/Cas9-targeted mutagenesis confer resistance to Rice tungro spherical virus. Plant Biotechnol. J. 2018, 16 (11), 1918-1927
Rice	Facilitate pathogen growth	CRISPR-Cas9	2019	Foster, A. J.; Martin-Urdiroz, M.; Yan, X.; Wright, H. S.; Soanes, D. M.; Talbot, N. J. CRISPR-Cas9 ribonucleoprotein-mediated co-editing and counterselection in the rice blast fungus. Sci. Rep. 2018, 8 (1), 1-12.
Rice	Disease facilitation	CRISPR-Cas9	2017	Nawaz, G.; Usman, B.; Peng, H.; Zhao, N.; Yuan, R.; Liu, Y.; Li, R Knockout of Pi21 by CRISPR/Cas9 and iTRAQ-Based Proteomic Analysis of Mutants Revealed New Insights into M. oryzae Resistance in Elite Rice Line. Genes 2020, 11 (7), 735. Li, S.; Shen, L.; Hu, P.; Liu, Q.; Zhu, X.; Qian, Q.; Wang, K.; Wang, Y. Developing disease-resistant thermosensitive male sterile rice by multiplex gene editing. J. Integr. Plant Biol. 2019, 61 (12), 1201-1205.
Rice	Disease facilitation	CRISPR-Cas9	2014	Wang, F.; Wang, C.; Liu, P.; Lei, C.; Hao, W.; Gao, Y.; Liu, Y.-G.; Zhao, K. Enhanced rice blast resistance by CRISPR/Cas9-targeted mutagenesis of the ERF transcription factor gene OsERF922. PLoS One 2016, 11 (4), e0154027.
Rice	Rice-Tungro- Virus resistance	CRISPR/Cas9 SDN1	2018	Macovei A, Sevilla NR, Cantos C, Jonson GB, Slamet-Loedin I, Čermák T, et al. Novel alleles of rice eIF4G generated by CRISPR/Cas9-targeted mutagenesis confer resistance to Rice tungro spherical virus. Plant Biotechnol J. 2018;47:417. doi:10.1111/pbi.12927.

Rice	Southern Rice Black-streaked Dwarf Virus resistance	CRISPR/Cas13a SDN1	2019	Zhang T, Zhao YL, Ye JJ, Cao X, Xu CH, Chen B, et al. Establishing CRISPR/Cas13a immune system conferring RNA virus resistance in both dicot and monocot plants. 1467- 7644. 2019;17:1185–7. doi:10.1111/pbi.13095.
Tomato	Broad spectrum	CRISPR/Cas9	2016	de Toledo Thomazella, D. P., Brail, Q., Dahlbeck, D., and Staskawicz, B. (2016). CRISPR-Cas9 mediated mutagenesis of a DMR6 ortholog in tomato confers broad-spectrum disease resistance. BioRxiv [Preprint]. doi: 10.1101/064824
Tomato	Powdery mildew	CRISPR/Cas9	2017	Nekrasov, V., Wang, C., Win, J., Lanz, C., Weigel, D., and Kamoun, S. (2017). Rapid generation of a transgene-free powdery mildew resistant tomato by genome deletion. Sci. Rep. 7:482. doi: 10.1038/s41598-017-00578-x
Tomato	Bacterial speck	CRISPR/Cas9	2019	Ortigosa, A., Gimenez-Ibanez, S., Leonhardt, N., and Solano, R. (2019). Design of a bacterial speck resistant tomato by CRISPR/Cas9-mediated editing of SlJAZ2. Plant Biotechnol. J. 17, 665–673. doi: 10.1111/pbi.13006
Tomato	Tomato yellow leaf curl virus	CRISPR/Cas9	2018	Tashkandi, M., Ali, Z., Aljedaani, F., Shami, A., and Mahfouz, M. M. (2018). Engineering resistance against tomato yellow leaf curl virus via the CRISPR/Cas9 system in tomato. Plant Signal. Behav. 13:e1525996. doi: 10.1080/15592324.2018.1525996
Tomato	Disease	CRISPR-Cas9	2020	Shu, P.; Li, Z.; Min, D.; Zhang, X.; Ai, W.; Li, J.; Zhou, J.; Li, Z.; Li, F.; Li, X. CRISPR/Cas9-Mediated SlMYC2 Mutagenesis Adverse to Tomato Plant Growth and MeJAInduced Fruit Resistance to Botrytis cinerea. J. Agric. Food Chem. 2020, 68 (20), 5529-

Tomato	Powdery mildew resistance	CRISPR/Cas9 SDN1	2017	Nekrasov V, Wang C, Win J, Lanz C, Weigel D, Kamoun S. Rapid generation of a transgene-free powdery mildew resistant tomato by genome deletion. Sci Rep. 2017;7:482. doi:10.1038/s41598-017-00578-x.
Tomato	Disease facilitation	CRISPR-Cas9	2020	Ortigosa, A.; Gimenez-Ibanez, S.; Leonhardt, N.; Solano, R. Design of a bacterial speck resistant tomato by CRISPR/Cas9-mediated editing of Sl JAZ 2. Plant Biotechnol. J. 2019, 17 (3), 665-673.
Tomato	Disease facilitation	CRISPR-Cas9	2020	Koseoglou, E. The study of SlPMR4 CRISPR/Cas9-mediated tomato allelic series for resistance against powdery mildew. Master thesis, Wageningen University and Research, Wageningen, 2017.
Tomato	Disease facilitation	CRISPR-Cas9	2020	Nekrasov, V.; Wang, C.; Win, J.; Lanz, C.; Weigel, D.; Kamoun, S. Rapid generation of a transgene-free powdery mildew resistant tomato by genome deletion. Sci. Rep. 2017, 7 (1), 1-6.
Tomato	leaf and fruit blotch disease resistance	CRISPR/Cas9 SDN1	2019	Ortigosa A, Gimenez-Ibanez S, Leonhardt N, Solano R. Design of a bacterial speck resistant tomato by CRISPR/Cas9-mediated editing of SlJAZ2. 1467-7644. 2019;17:665– 73. doi:10.1111/pbi.13006.
Tomato	Multiple resistance e.g. P. syringae, P. capsici und Xanthomonas spp.	CRISPR/Cas9 SDN1	2016	Toledo Thomazella DP de, Brail Q, Dahlbeck D, Staskawicz BJ. CRISPR-Cas9 mediated mutagenesis of a DMR6 ortholog in tomato confers broad-spectrum disease resistance. 2016:1–23. doi:10.1101/064824.
Tomato	Tomato Yellow Leaf Virus resistance	CRISPR/Cas9 SDN1	2017	Mahfouz M, Tashkandi M, Ali Z, Aljedaani F, Shami A. Engineering resistance against Tomato yellow leaf curl virus via the CRISPR/Cas9 system in tomato 2017. doi:10.1101/237735.

Wheat	Powdery mildew	TALENS, CRISPR/Cas9	2014	Wang, Y., Cheng, X., Shan, Q., Zhang, Y., Liu, J., Gao, C., et al. (2014). Simultaneous editing of three homoeoalleles in hexaploid bread wheat confers heritable resistance to powdery mildew. Nat. Biotechnol. 32, 947–951. doi: 10.1038/nbt.2969
Wheat	Powdery mildew resistance	TALENs SDN1	2014	Wang Y, Cheng X, Shan Q, Zhang Y, Liu J, Gao C, Qiu J-L. Simultaneous editing of three homoeoalleles in hexaploid bread wheat confers heritable resistance to powdery mildew. Nat Biotechnol. 2014;32:947–51. doi:10.1038/nbt.2969.
Wheat	Powdery mildew resistance	CRISPR/Cas9 SDN1	2017	Zhang Y, Bai Y, Wu G, Zou S, Chen Y, Gao C, Tang D. Simultaneous modification of three homoeologs of TaEDR1 by genome editing enhances powdery mildew resistance in wheat. Plant J. 2017;91:714–24. doi:10.1111/tpj.13599.
Wheat	Disease facilitation	CRISPR-Cas9	2019	Zhang, Y.; Bai, Y.; Wu, G.; Zou, S.; Chen, Y.; Gao, C.; Tang, D. Simultaneous modification of three homoeologs of Ta EDR 1 by genome editing enhances powdery mildew resistance in wheat. Plant J. 2017, 91 (4), 714-724.
