# Supplementary material for: Genome editing techniques in plants: a comprehensive review and future prospects toward zero hunger
Source: GM Crops Food. 2022 Feb 9;12(2):601–15. doi: 10.1080/21645698.2021.2021724 (PMC9208631; doi:10.1080/21645698.2021.2021724)
Supplement: Supplemental Material [file KGMC_A_2021724_SM8963.zip › Table_S3.pdf]

Table S3: Summary of role of CRISPR system in breeding improvement

Trait	Plant	Gene Function	Technique	Year	References
Grain yield	Barley	cytokinin metabolism	CRISPR-Cas9	2015	Holubová, K.; Hensel, G.; Vojta, P.; Tarkowski, P.; Bergougnoux, V.; Galuszka, P. Modification of barley plant productivity through regulation of cytokinin content by reversegenetics approaches. Front. Plant Sci. 2018, 9, 1676.
	Rice	Negatively regulates the grain number	CRISPR-Cas9	2018	Shen, L.; Wang, C.; Fu, Y.; Wang, J.; Liu, Q.; Zhang, X.; Yan, C.; Qian, Q.; Wang, K. QTL editing confers opposing yield performance in different rice varieties. J. Integr. Plant Biol. 2018, 60 (2), 89-93.<br><br>Huang, L.; Zhang, R.; Huang, G.; Li, Y.; Melaku, G.; Zhang, S.; Chen, H.; Zhao, Y.; Zhang, J.; Zhang, Y. Developing superior alleles of yield genes in rice by artificial mutagenesis using the CRISPR/Cas9 system. Crop J. 2018, 6 (5), 475-481.
	Rice	Negatively regulates root growth, tiller number, and yield	CRISPR-Cas9	2018	Wang, C.; Wang, G.; Gao, Y.; Lu, G.; Habben, J. E.; Mao, G.; Chen, G.; Wang, J.; Yang, F.; Zhao, X. A cytokinin-activation enzyme-like gene improves grain yield under various field conditions in rice. Plant Mol. Biol. 2020, 102 (4), 373-388.
	Rice	Negatively regulates yield parameters	CRISPR-Cas9	2019	Zeng, Y.; Wen, J.; Zhao, W.; Wang, Q.; Huang, W. Rational Improvement of Rice Yield and Cold Tolerance by Editing the Three Genes OsPIN5b, GS3, and OsMYB30 With the CRISPR–Cas9 System. Front. Plant Sci. 2020, 10, 1663.

	Rice	inflorescence architecture and plant height	CRISPR-Cas9	2016	Huang, L.; Zhang, R.; Huang, G.; Li, Y.; Melaku, G.; Zhang, S.; Chen, H.; Zhao, Y.; Zhang, J.; Zhang, Y. Developing superior alleles of yield genes in rice by artificial mutagenesis using the CRISPR/Cas9 system. Crop J. 2018, 6 (5), 475-481.
	Wheat	Negatively regulates grain number per spikelet	CRISPR-Cas9	2020	Zhang, Z.; Hua, L.; Gupta, A.; Tricoli, D.; Edwards, K. J.; Yang, B.; Li, W. Development of an Agrobacterium-delivered CRISPR/Cas9 system for wheat genome editing. Plant Biotechnol. J. 2019, 17 (8), 1623-1635.
	Wheat	inflorescence architecture and plant height regulator, lipoxygenase, negative regulator of grain weight	CRISPR-Cas9	2018	Zhang, Z.; Hua, L.; Gupta, A.; Tricoli, D.; Edwards, K. J.; Yang, B.; Li, W. Development of an Agrobacterium-delivered CRISPR/Cas9 system for wheat genome editing. Plant Biotechnol. J. 2019, 17 (8), 1623-1635.<br><br>Zhang, Y.; Gao, C. Recent advances in DNA-free editing and precise base editing in plants. Emerging Top. Life Sci. 2017, 1 (2), 161-168.
Growth performance	Cabbage	Self-incompatibility, male sterility	CRISPR/Cas9 SDN1	2019	Ma CF, Zhu CZ, Zheng M, Liu MC, Zhang DJ, Liu BL, et al. CRISPR/Cas9-mediated multiple gene editing in Brassica oleracea var. capitata using the endogenous tRNAprocessing system. Hortic Res-England. 2019;6.
	Canola	Early flowering	CRISPR/Cas9 SDN1	2018	Jiang L, Li DH, Jin L, Ruan Y, Shen WH, Liu CL. Histone lysine methyltransferases BnaSDG8.A and BnaSDG8.C are involved in the floral transition in Brassica napus. Plant J. 2018;95:672–85. doi:10.1111/tpj.13978.

Cotton	Increased root growth under high- and low N- conditions	CRISPR/Cas9 SDN1	2017	Wang Y, Meng Z, Liang C, Meng Z, Wang Y, Sun G, et al. Increased lateral root formation by CRISPR/Cas9-mediated editing of arginase genes in cotton. Sci China Life Sci. 2017;60:524–7. doi:10.1007/s11427-017-9031-y.
Cucumber	Only female flowers	CRISPR/Cas9 SDN1	2017	Hu B, Li D, Liu X, Qi J, Gao D, Zhao S, et al. Engineering Non-transgenic Gynoecious Cucumber Using an Improved Transformation Protocol and Optimized CRISPR/Cas9 System. Mol Plant. 2017;10:1575–8. doi:10.1016/j.molp.2017.09.005.
Dandelion	Higher biomass of roots, taproots, increased rubber and inulin content	CRISPR/Cas9	2019	Wieghaus A, Prufer D, Gronover CS. Loss of function mutation of the Rapid Alkalinization Factor (RALF1)-like peptide in the dandelion Taraxacum koksaghyz entails a high-biomass taproot phenotype. Plos One. 2019;14.
Japanese morning glory	increased flowering time	CRISPR/Cas9 SDN1	2018	Shibuya K, Watanabe K, Ono M. CRISPR/Cas9-mediated mutagenesis of the EPHEMERAL1 locus that regulates petal senescence in Japanese morning glory. Plant Physiol Biochem 2018. doi:10.1016/j.plaphy.2018.04.036.
Japanese morning glory	Changed flower color	CRISPR/Cas9 SDN2	2017	Watanabe K, Oda-Yamamizo C, Sage-Ono K, Ohmiya A, Ono M. Alteration of flower colour in Ipomoea nil through CRISPR/Cas9-mediated mutagenesis of carotenoid cleavage dioxygenase 4. Transgenic Res. 2017;27:25–38. doi:10.1007/s11248-017-0051-0

Kiwi	Compact growth, early flowering	CRISPR/Cas9 SDN1	2019	Varkonyi-Gasic E, Wang TC, Voogd C, Jeon S, Drummond RSM, Gleave AP, Allan AC. Mutagenesis of kiwifruit CENTRORADIALIS-like genes transforms a climbing woody perennial with long juvenility and axillary flowering into a compact plant with rapid terminal flowering. 1467-7644. 2019;17:869–80. doi:10.1111/pbi.13021.
Maize	male sterility	CRISPR/Cas9 SDN3	2018	Chen RR, Xu QL, Liu Y, Zhang JJ, Ren DT, Wang GY, Liu YJ. Generation of Transgene-Free Maize Male Sterile Lines Using the CRISPR/Cas9 System. Front Plant Sci. 2018;9.
Maize	male sterility	CRISPR/Cas9 SDN1	2019	hiou WY, Kawamoto T, Himi E, Rikiishi K, Sugimoto M, Hayashi-Tsugane M, et al. LARGE GRAIN Encodes a Putative RNA-Binding Protein that Regulates Spikelet Hull Length in Rice. Plant Cell Physiol. 2019;60:503–15. doi:10.1093/pcp/pcz014.
Maize	Haploid induction	TALENs SDN1	2017	Kelliher T, Starr D, Richbourg L, Chintamanani S, Delzer B, Nuccio ML, et al. MATRILINEAL, a sperm-specific phospholipase, triggers maize haploid induction. Nature. 2017;542:105–9. doi:10.1038/nature20827.37
Maize	Haploid induction	CRISPR/Cas9 SDN1	2019	Kelliher T, Starr D, Su XJ, Tang GZ, Chen ZY, Carter J, et al. One-step genome editing of elite crop germplasm during haploid induction. Nat Biotechnol. 2019;37:287-+. doi:10.1038/s41587-019-0038-x.
Maize	male sterility	CRISPR/Cas9 SDN2	2017	Li J, Zhang H, Si X, Tian Y, Chen K, Liu J, et al. Generation of thermosensitive malesterile maize by targeted knockout of the ZmTMS5 gene. J Genet Genomics. 2017;44:465–8. doi:10.1016/j.jgg.2017.02.002.

Maize	male sterility	CRISPR/Cas9 SDN1	2018	Xie K, Wu S, Li Z, Zhou Y, Zhang D, Dong Z, et al. Map-based cloning and characterization of Zea mays male sterility33 (ZmMs33) gene, encoding a glycerol-3- phosphate acyltransferase. Theor Appl Genet. 2018;131:1363–78. doi:10.1007/s00122- 018-3083-9.
Maize	Haploid induction	CRISPR/Cas9 SDN2	2019	Zhong Y, Liu C, Qi X, Jiao Y, Wang D, Wang Y, et al. Mutation of ZmDMP enhances haploid induction in maize 2019. doi:10.1038/s41477-019-0443-7.
Millet	bushy plants	CRISPR/Cas9 SDN1	2018	Liu Y, Merrick P, Zhang Z, Ji C, Yang B, Fei S-z. Targeted mutagenesis in tetraploid switchgrass (Panicum virgatum L.) using CRISPR/Cas9. Plant Biotechnol J. 2018;16:381–93. doi:10.1111/pbi.12778.
Petunia	increased flowering time of individual flowers	CRISPR/Cas9 SDN1	2019	Xu J, Kang BC, Naing AH, Bae SJ, Kim JS, Kim H, Kim CK. CRISPR/Cas9-mediated editing of 1-aminocyclopropane-1-carboxylate oxidase1 (ACO1) enhances Petunia flower longevity. 1467-7644 2019. doi:10.1111/pbi.13197.
Physalis	compact growth, increased flower production, bigger fruits	CRISPR/Cas9 SDN1	2018	Lemmon ZH, Reem NT, Dalrymple J, Soyk S, Swartwood KE, Rodriguez-Leal D, et al. Rapid improvement of domestication traits in an orphan crop by genome editing. Nat Plants. 2018;4:766–70. doi:10.1038/s41477-018-0259-x.
Potato	Self-incompatibility	CRISPR/Cas9 SDN1	2018	Ye MW, Peng Z, Tang D, Yang ZM, Li DW, Xu YM, et al. Generation of selfcompatible diploid potato by knockout of S-RNase. Nat Plants. 2018;4:651–4. doi:10.1038/s41477-018-0218-6.

Rice	better grain productivity and growth	CRISPR-Cas9	2019	Miao, C.; Xiao, L.; Hua, K.; Zou, C.; Zhao, Y.; Bressan, R. A.; Zhu, J.-K. Mutations in a subfamily of abscisic acid receptor genes promote rice growth and productivity. Proc. Natl. Acad. Sci. U.S.A. 2018, 115 (23), 6058-6063.
Rice	Male sterility	CRISPR/Cas9 SDN1	2019	Barman HN, Sheng ZH, Fiaz S, Zhong M, Wu YW, Cai YC, et al. Generation of a new thermo-sensitive genic male sterile rice line by targeted mutagenesis of TMS5 gene through CRISPR/Cas9 system. Bmc Plant Biol. 2019;19.
Rice	improved tillering, reduced plant height	CRISPR/Cas9 SDN1	2018	Butt H, Jamil M, Wang JY, Al-Babili S, Mahfouz M. Engineering plant architecture via CRISPR/Cas9-mediated alteration of strigolactone biosynthesis. Bmc Plant Biol. 2018;18. 27
Rice	Early flowering	CRISPR/Cas9 SDN2	2019	Cui Y, Zhu MM, Xu ZJ, Xu Q. Assessment of the effect of ten heading time genes on reproductive transition and yield components in rice using a CRISPR/Cas9 system. Theor Appl Genet. 2019;132:1887–96. doi:10.1007/s00122-019-03324-1.
Rice	Dormancy regulation, stomata development, improved plant growth, abiotic Stress tolerance and senescence	CRISPR/Cas9 SDN1	2018	Huang Y, Guo Y, Liu Y, Zhang F, Wang Z, Wang H, et al. 9-cis-Epoxycarotenoid Dioxygenase 3 Regulates Plant Growth and Enhances Multi-Abiotic Stress Tolerance in Rice. Front. Plant Sci. 2018;9:1248. doi:10.3389/fpls.2018.00162.

Rice	Asexual reproduction	CRISPR/Cas9 SDN1	2019	Khanday I, Skinner D, Yang B, Mercier R, Sundaresan V. A male-expressed rice embryogenic trigger redirected for asexual propagation through seeds. Nature. 2019;565:91-+. doi:10.1038/s41586-018-0785-8.
Rice	Male sterility	CRISPR/Cas9 SDN1	2016	Lee S-K, Eom J-S, Hwang S-K, Shin D, An G, Okita TW, Jeon J-S. Plastidic phosphoglucomutase and ADP-glucose pyrophosphorylase mutants impair starch synthesis in rice pollen grains and cause male sterility. J Exp Bot. 2016;67:5557–69. doi:10.1093/jxb/erw324.
Rice	Male sterility	CRISPR/Cas9 SDN1	2016	Li Q, Zhang D, Chen M, Liang W, Wei J, Qi Y, Yuan Z. Development of japonica Photo-Sensitive Genic Male Sterile Rice Lines by Editing Carbon Starved Anther Using CRISPR/Cas9. J Genet Genomics. 2016;43:415–9. doi:10.1016/j.jgg.2016.04.011.
Rice	Early ripening	CRISPR/Cas9 SDN1	2017	Li X, Zhou W, Ren Y, Tian X, Lv T, Wang Z, et al. High-efficiency breeding of earlymaturing rice cultivars via CRISPR/Cas9-mediated genome editing. J Genet Genomics. 2017;44:175–8. doi:10.1016/j.jgg.2017.02.001.
Rice	Larger plants, improved tillering, upright panicles, increased biomass	CRISPR/Cas9 SDN1	2018	Liao Y, Bai Q, Xu P, Wu T, Guo D, Peng Y, et al. Mutation in Rice Abscisic Acid2 Results in Cell Death, Enhanced Disease-Resistance, Altered Seed Dormancy and Development. Front. Plant Sci. 2018;9:1248. doi:10.3389/fpls.2018.00405.
Rice	reduced plant height	BE SDN1	2017	Lu Y, Zhu J-K. Precise Editing of a Target Base in the Rice Genome Using a Modified CRISPR/Cas9 System. Mol Plant. 2017;10:523–5. doi:10.1016/j.molp.2016.11.013.

Rice	(increased surface)	SDN1	2019	Qu L, Lin LB, Xue HW. Rice miR394 suppresses leaf inclination through targeting an Fbox gene, LEAF INCLINATION 4. J Integr Plant Biol. 2019;61:406–16. doi:10.1111/jipb.12713.
Rice	Male sterility	CRISPR/Cas9 SDN1	2019	Shen L, Dong GJ, Zhang Y, Hu GC, Zhang Q, Hu GL, et al. Rapid Creation of New Photoperiod-/Thermo-Sensitive Genic Male-Sterile Rice Materials by CRISPR/Cas9 System. Rice Sci. 2019;26:129–32. doi:10.1016/j.rsci.2018.12.006.
Rice	Male sterility	CRISPR/Cas9 SDN2	2018	Shi QS, Wang KQ, Li YL, Zhou L, Xiong SX, Han Y, et al. OsPKS1 is required for sexine layer formation, which shows functional conservation between rice and Arabidopsis. 0168-9452. 2018;277:145–54. doi:10.1016/j.plantsci.2018.08.009.
Rice	Heterozygosity, clonal Seed propagation	CRISPR/Cas9	2019	Wang C, Liu Q, Shen Y, Hua YF, Wang JJ, Lin JR, et al. Clonal seeds from hybrid rice by simultaneous genome engineering of meiosis and fertilization genes. Nat Biotechnol. 2019;37:283-+. doi:10.1038/s41587-018-0003-0.
Rice	Early flowering	CRISPR/Cas9 SDN1	2018	Zeng LP, Liu X, Zhou ZZ, Li DY, Zhao XF, Zhu LH, et al. Identification of a G2-like transcription factor, OsPHL3, functions as a negative regulator of flowering in rice by coexpression and reverse genetic analysis. Bmc Plant Biol. 2018;18.
Rice	Male sterility	CRISPR/Cas9 SDN2	2016	Zhou H, He M, Li J, Chen L, Huang Z, Zheng S, et al. Development of Commercial Thermo-sensitive Genic Male Sterile Rice Accelerates Hybrid Rice Breeding Using the CRISPR/Cas9-mediated TMS5 Editing System. Sci Rep. 2016;6:1–12. doi:10.1038/srep37395
Rice	Red colored rice	CRISPR/Cas9 SDN1	2019	Zhu Y, Lin Y, Chen S, Liu H, Chen Z, Fan M, et al. CRISPR/Cas9-mediated functional recovery of the recessive rc allele to develop red rice. 1467-7644 2019. doi:10.1111/pbi.13125.

Rice	Male sterility	CRISPR/Cas9 SDN2	2017	Zou T, Xiao Q, Li W, Luo T, Yuan G, He Z, et al. OsLAP6/OsPKS1, an orthologue of Arabidopsis PKSA/LAP6, is critical for proper pollen exine formation. Rice (N Y). 2017;10:615. doi:10.1186/s12284-017-0191-0.
Soybean	late flowering	CRISPR/Cas9 SDN2	2018	Cai YP, Chen L, Sun S, Wu CX, Yao WW, Jiang BJ, et al. CRISPR/Cas9-Mediated Deletion of Large Genomic Fragments in Soybean. Int J Mol Sci. 2018;19.
Soybean	Changed petiole length	CRISPR/Cas9 SDN1	NA	Petiol length. APHIS-Am I Regulated. https://www.aphis.usda.gov/biotechnology/downloads/reg_loi/19-077-01_air_inquiry.pdf.
Soybean	late flowering under short-day conditions improved pod- and seed set/Plant	CRISPR/Cas9 SDN1	2019	Cai Y, Wang L, Chen L, Wu T, Liu L, Sun S, et al. Mutagenesis of GmFT2a and GmFT5a mediated by CRISPR/Cas9 contribute for expanding the regional adaptability of soybean. 1467-7644 2019. doi:10.1111/pbi.13199.
Tomato	Yellow fruits	CRISPR/Cas9 SDN1	2018	D'Ambrosio C, Stigliani AL, Giorio G. CRISPR/Cas9 editing of carotenoid genes in tomato. Transgenic Res. 2018;27:367–78. doi:10.1007/s11248-018-0079-9.
Tomato	Orange fruits	CRISPR/Cas9 SDN3	2018	Dahan-Meir T, Filler-Hayut S, Melamed-Bessudo C, Bocobza S, Czosnek H, Aharoni A, Levy AA. Efficient in planta gene targeting in tomato using geminiviral replicons and the CRISPR/Cas9 system. The Plant Journal. 2018;95:5–16. doi:10.1111/tpj.13932.
Tomato	Pink fruits	CRISPR/Cas9 SDN1	2018	Deng L, Wang H, Sun C, Li Q, Jiang H, Du M, et al. Efficient generation of pink-fruited tomatoes using CRISPR/Cas9 system. J Genet Genomics. 2018;45:51–4. doi:10.1016/j.jgg.2017.10.002.

Tomato	Yellow fruits	CRISPR/Cas9 SDN1	2017	Filler Hayut S, Melamed Bessudo C, Levy AA. Targeted recombination between homologous chromosomes for precise breeding in tomato. Nat Commun. 2017;8:15605. doi:10.1038/ncomms15605.
Tomato	Bigger seedlings	TALENs SDN1	2014	Lor VS, Starker CG, Voytas DF, Weiss D, Olszewski NE. Targeted mutagenesis of the tomato PROCERA gene using transcription activator-like effector nucleases. Plant Physiol. 2014;166:1288–91. doi:10.1104/pp.114.247593.28
Tomato	Early flowering	CRISPR/Cas9 SDN1	2017	Soyk S, Müller NA, Park SJ, Schmalenbach I, Jiang K, Hayama R, et al. Variation in the flowering gene SELF PRUNING 5G promotes day-neutrality and early yield in tomato. Nat Genet. 2017;49:162–8. doi:10.1038/ng.3733.
Tomato	Dwarfism	CRISPR/Cas9 SDN1	2019	Tomlinson L, Yang Y, Emenecker R, Smoker M, Taylor J, Perkins S, et al. Using CRISPR/Cas9 genome editing in tomato to create a gibberellin-responsive dominant dwarf DELLA allele. 1467-7644. 2019;17:132–40. doi:10.1111/pbi.12952.
Tomato	Easier separation of the fruit from the stalk	CRISPR/Cas9 SDN1	2018	United States Department of Agriculture (USDA). 2018. https://www.aphis.usda.gov/biotechnology/downloads/reg_loi/18-051-01_air_response_signed.pdf. Accessed 25 Aug 2018.
Torenia	Changed flower color	CRISPR/Cas9 SDN1	2018	Nishihara M, Higuchi A, Watanabe A, Tasaki K. Application of the CRISPR/Cas9 system for modification of flower color in Torenia fournieri. BMC Plant Biol. 2018;18:331. doi:10.1186/s12870-018-1539-3.

	Wheat	Male sterility	CRISPR/Cas9 SDN2	2019	Okada A, Arndell T, Borisjuk N, Sharma N, Watson-Haigh NS, Tucker EJ, et al. CRISPR/Cas9-mediated knockout of Ms1 enables the rapid generation of male-sterile hexaploid wheat lines for use in hybrid seed production 2019. doi:10.1111/pbi.13106.
	Wheat	Male sterility	CRISPR/Cas9 SDN1	2018	Singh M, Kumar M, Albertsen MC, Young JK, Cigan AM. Concurrent modifications in the three homeologs of Ms45 gene with CRISPR-Cas9 lead to rapid generation of male sterile bread wheat (Triticum aestivum L.). Plant Mol Biol. 2018;97:371–83. doi:10.1007/s11103-018-0749-2.
	Wild strawberry	Faster seedling development	SDN1	2018	Zhou J, Wang G, Liu Z. Efficient genome editing of wild strawberry genes, vector development and validation. Plant Biotechnol J. 2018;166:1292. doi:10.1111/pbi.12922.
Plant height	-	Control plant height	CRISPR/Cas9	2017	Lu, Y.; Zhu, J.-K. Precise editing of a target base in the rice genome using a modified CRISPR/Cas9 system. Mol Plant. 2017, 10 (3), 523-525.
Root architecture	Rice	root growth angle	CRISPR/Cas9	2018	Kitomi, Y.; Hanzawa, E.; Kuya, N.; Inoue, H.; Hara, N.; Kawai, S.; Kanno, N.; Endo, M.; Sugimoto, K.; Yamazaki, T. Root angle modifications by the DRO1 homolog improve rice yields in saline paddy fields. Proc. Natl. Acad. Sci. U.S.A. 2020, 117 (35), 21
Yield increase	Canola	Scatter resistance	CRISPR/Cas9 SDN1	2017	Braatz J, Harloff H-J, Mascher M, Stein N, Himmelbach A, Jung C. CRISPR-Cas9 Targeted Mutagenesis Leads to Simultaneous Modification of Different Homoeologous Gene Copies in Polyploid Oilseed Rape (Brassica napus). Plant Physiol. 2017;174:9. doi:10.1104/pp.17.00426.

Canola	Increased seed number/pod, higher grain weight	CRISPR/Cas9 SDN1	2018	Yang Y, Zhu KY, Li HL, Han SQ, Meng QW, Khan SU, et al. Precise editing of CLAVATA genes in Brassica napus L. regulates multilocular silique development. 1467- 7644. 2018;16:1322–35. doi:10.1111/pbi.12872.
Lettuce	Increased germination under higher temp.	CRISPR/Cas9 SDN1	2018	Bertier LD, Ron M, Huo H, Bradford KJ, Britt AB, Michelmore RW. High-Resolution Analysis of the Efficiency, Heritability, and Editing Outcomes of CRISPR/Cas9-Induced Modifications of NCED4 in Lettuce (Lactuca sativa). G3 (Bethesda). 2018;8:1513–21. doi:10.1534/g3.117.300396.
Rice	Higher Grain size and weight / Thousand seed weight	CRISPR/Cas9 SDN1	2019	Chiou WY, Kawamoto T, Himi E, Rikiishi K, Sugimoto M, Hayashi-Tsugane M, et al. LARGE GRAIN Encodes a Putative RNA-Binding Protein that Regulates Spikelet Hull Length in Rice. Plant Cell Physiol. 2019;60:503–15. doi:10.1093/pcp/pcz014.
Rice	Higher Grain size and weight / Thousand seed weight	CRISPR/Cas9 SDN1	2018	Hu Z, Lu S-J, Wang M-J, He H, Le Sun, Wang H, et al. A Novel QTL q TGW3 Encodes the GSK3/SHAGGY-Like Kinase OsGSK5/OsSK41 that Interacts with OsARF4 to Negatively Regulate Grain Size and Weight in Rice. Mol Plant. 2018;11:736–49. doi:10.1016/j.molp.2018.03.005.
Rice	Higher Grain size and weight / Thousand seed weight	CRISPR/Cas9 SDN1	2017	Ji X, Li F, Yan Y, Sun HZ, Zhang J, Li JZ, et al. CRISPR/Cas9 System-Based Editing of Phytochrome-Interacting Factor OsPIL15 2017. doi:10.3864/j.issn.0578- 1752.2017.15.002.
Rice	Regulation of pollen growth	CRISPR/Cas9 SDN1	2016	Liu L, Zheng C, Kuang B, Wei L, Yan L, Wang T. Receptor-Like Kinase RUPO Interacts with Potassium Transporters to Regulate Pollen Tube Growth and Integrity in Rice. PLoS Genet. 2016;12:e1006085. doi:10.1371/journal.pgen.1006085.

Rice	Improved N-efficiency	CRISPR/Cas9 SDN1	2018	Lu K, Wu B, Wang J, Zhu W, Nie H, Qian J, et al. Blocking amino acid transporter OsAAP3 improves grain yield by promoting outgrowth buds and increasing tiller number in rice. Plant Biotechnol J. 2018;50:1416. doi:10.1111/pbi.12907.
Rice	Higher Grain size and weight / Thousand seed weight	CRISPR/Cas9 SDN2	2019	Ma XS, Feng FJ, Zhang Y, Elesawi IE, Xu K, Li TF, et al. A novel rice grain size gene OsSNB was identified by genome-wide association study in natural population. Plos Genet. 2019;15.
Rice	Higher Grain size and weight / Thousand seed weight	CRISPR/Cas9 SDN2	2019	Miao J, Yang ZF, Zhang DP, Wang YZ, Xu MB, Zhou LH, et al. Mutation of RGG2, which encodes a type B heterotrimeric G protein gamma subunit, increases grain size and yield production in rice. 1467-7644. 2019;17:650–64. doi:10.1111/pbi.13005.
Rice	Increased seed number/panicle	CRISPR/Cas9 SDN1	2016	Shen L, Wang C, Fu Y, Wang J, Liu Q, Zhang X, et al. QTL editing confers opposing yield performance in different rice varieties. J Integr Plant Biol 2016. doi:10.1111/jipb.12501. 26
Rice	Higher Grain size and weight / Thousand seed weight	CRISPR/Cas9 SDN1	2017	Shen Lan, Li Jian, Fu Yaping, Wang Junjie, Hua Yufeng, Jiao Xiaozhen, Yan Changjie, Wang Kejian. Orientation Improvement of Grain Length and Grain Number in Rice by Using CRISPR/Cas9 System 2017. doi:10.16819/j.1001-7216.2017.7029.
Rice	Increased seed number/panicle	CRISPR/Cas9 SDN1	2019	Wang J, Wu BW, Lu K, Wei Q, Qian JJ, Chen YP, Fang ZM. The Amino Acid Permease 5 (OsAAP5) Regulates Tiller Number and Grain Yield in Rice. 0032-0889. 2019;180:1031–45. doi:10.1104/pp.19.00034.
Rice	Longer penicles	CRISPR/Cas9 SDN1	2016	Xu R, Yang Y, Qin R, Li H, Qiu C, Li L, et al. Rapid improvement of grain weight via highly efficient CRISPR/Cas9-mediated multiplex genome editing in rice. J Genet Genomics. 2016;43:529–32. doi:10.1016/j.jgg.2016.07.003.

Rice	Grain yield, Regulation of seed-development	CRISPR/Cas9 SDN1	2017	Yuan J, Chen S, Jiao W, Wang L, Wang L, Ye W, et al. Both maternally and paternally imprinted genes regulate seed development in rice. New Phytol. 2017;216:373–87. doi:10.1111/nph.14510.
Rice	Higher Grain size and weight / Thousand seed weight	CRISPR/Cas9 SDN1	2019	Zhou JP, Xin XH, He Y, Chen HQ, Li Q, Tang X, et al. Multiplex QTL editing of grainrelated genes improves yield in elite rice varieties. Plant Cell Rep. 2019;38:475–85. doi:10.1007/s00299-018-2340-3.
Tomato	Fast ripping fruits	CRISPR/Cas9 SDN1	2015	Ito Y, Nishizawa-Yokoi A, Endo M, Mikami M, Toki S. CRISPR/Cas9-mediated mutagenesis of the RIN locus that regulates tomato fruit ripening. Biochem Biophys Res Commun. 2015;467:76–82. doi:10.1016/j.bbrc.2015.09.117.
Tomato	Strongly branched inflorescences and formation of many flowers	CRISPR/Cas9 SDN1	2017	Rodríguez-Leal D, Lemmon ZH, Man J, Bartlett ME, Lippman ZB. Engineering Quantitative Trait Variation for Crop Improvement by Genome Editing. Cell. 2017;171:470-480.e8. doi:10.1016/j.cell.2017.08.030.
Tomato	Fast ripping fruits	CRISPR/Cas9 SDN1	2019	Wang RF, Tavano ECD, Lammers M, Martinelli AP, Angenent GC, Maagd RA de. Reevaluationof transcription factor function in tomato fruit development and ripening with CRISPR/Cas9-mutagenesis. Sci Rep-Uk. 2019;9.
Wheat	Larger grains,higher grain weight, higher thousand-grain weight	CRISPR/Cas9 SDN1	2018	Zhang Y, Li D, Zhang D, Zhao X, Cao X, Dong L, et al. Analysis of the functions of TaGW2 homoeologs in wheat grain weight and protein content traits. The Plant Journal. 2018;94:857–66. doi:10.1111/tpj.13903.

		Wheat	Higher grain number per ear,increased grain weight per ear	CRISPR/Cas9 SDN1	2019	Zhang Z, Hua L, Gupta A, Tricoli D, Edwards KJ, Yang B, Li W. Development of an Agrobacterium-delivered CRISPR/Cas9 system for wheat genome editing 2019. doi:10.1111/pbi.13088.
		Wild Tomato	changed fruit shape, compact growth, increased fruit number, larger fruits, increased constitutional ingredients (higher Lycopincontent)	CRISPR/Cas9 SDN2	2018	Zsögön A, ?ermák T, Naves ER, Notini MM, Edel KH, Weinl S, et al. De novo domestication of wild tomato using genome editing 2018. doi:10.1038/nbt.4272.
quality	Cooking and eating quality	Maize	amylose synthesis	CRISPR/Cas9	2017	Gao, H.; Gadlage, M. J.; Lafitte, H. R.; Lenderts, B.; Yang, M.; Schroder, M.; Farrell, J.; Snopek, K.; Peterson, D.; Feigenbutz, L. Superior field performance of waxy corn engineered using CRISPR–Cas9. Nat. Biotechnol. 2020, 38 (5), 579-581.
		Potato	amylose synthesis	CRISPRCas9 and Base Editing	2020	Veillet, F.; Chauvin, L.; Kermarrec, M.-P.; Sevestre, F.; Merrer, M.; Terret, Z.; Szydlowski, N.; Devaux, P.; Gallois, J.-L.; Chauvin, J.-E. The Solanum tuberosum GBSSI gene: a target for assessing gene and base editing in tetraploid potato. Plant Cell Rep. 2019, 38 (9), 1065-1080

Rice	amylose synthesis	CRISPR/Cas9	2019	Huang, L.; Li, Q.; Zhang, C.; Chu, R.; Gu, Z.; Tan, H.; Zhao, D.; Fan, X.; Liu, Q. Creating novel Wx alleles with fine-tuned amylose levels and improved grain quality in rice by promoter editing using CRISPR/Cas9 system. Plant Biotechnol. J. 2020, 18 (1
Rice	amylose synthesis	CRISPR/Cas9	2019	Khan, M. S. S.; Basnet, R.; Ahmed, S.; Bao, J.; Shu, Q. Mutations of OsPLDa1 Increase Lysophospholipid Content and Enhance Cooking and Eating Quality in Rice. Plants 2020, 9 (3), 390.
Rice	GBSS (amylose synthesis)	CRISPR/Cas9	2020	Ma, X.; Zhang, Q.; Zhu, Q.; Liu, W.; Chen, Y.; Qiu, R.; Wang, B.; Yang, Z.; Li, H.; Lin, Y. A robust CRISPR/Cas9 system for convenient, high-efficiency multiplex genome editing in monocot and dicot plants. Mol Plant. 2015, 8 (8), 1274-1284.
Rice	AGPase (starch biosynthesis)	CRISPR/Cas9	2021	Pérez, L.; Soto, E.; Villorbina, G.; Bassie, L.; Medina, V.; Muñoz, P.; Capell, T.; Zhu, C.; Christou, P.; Farré, G. CRISPR/Cas9-induced monoallelic mutations in the cytosolic AGPase large subunit gene APL2 induce the ectopic expression of APL2 and the corresponding small subunit gene APS2b in rice leaves. Transgenic Res. 2018, 27 (5), 423439
Rice	Betaine aldehyde dehydrogenase (fragrant rice)	CRISPR/Cas9	2020	Shufen, C.; Yicong, C.; Baobing, F.; Guiai, J.; Zhonghua, S.; Ju, L.; Shaoqing, T.; Jianlong, W.; Peisong, H.; Xiangjin, W. Editing of rice isoamylase gene ISA1 provides insights into its function in starch formation. Rice Sci. 2019, 26 (2), 77-87.

	Rice	Starch (isoamylasetype) debranching enzymes	CRISPR/Cas9	2020	Sun, Y.; Jiao, G.; Liu, Z.; Zhang, X.; Li, J.; Guo, X.; Du, W.; Du, J.; Francis, F.; Zhao, Y. Generation of high-amylose rice through CRISPR/Cas9-mediated targeted mutagenesis of starch branching enzymes. Front. Plant Sci. 2017, 8, 298.
	Rice	Amino acid transporter controlling grain protein content (GPC)	CRISPR/Cas9	2019	Wang, S.; Yang, Y.; Guo, M.; Zhong, C.; Yan, C.; Sun, S. Targeted mutagenesis of amino acid transporter genes for rice quality improvement using the CRISPR/Cas9 system. Crop J. 2020, 8 (3), 457-464.
	Rice	amylose synthesis	CRISPR/Cas9	2020	Zeng, D.; Liu, T.; Ma, X.; Wang, B.; Zheng, Z.; Zhang, Y.; Xie, X.; Yang, B.; Zhao, Z.; Zhu, Q. Quantitative regulation of Waxy expression by CRISPR/Cas9-based promoter and 5'UTR-intron editing improves grain quality in rice. Plant Biotechnol. J. 2020,
	Rice	Starchbranching enzyme	CRISPR/Cas9	2020	Zhang, J.; Zhang, H.; Botella, J. R.; Zhu, J. K. Generation of new glutinous rice by CRISPR/Cas9-targeted mutagenesis of the Waxy gene in elite rice varieties. J. Integr. Plant Biol. 2018, 60 (5), 369-375.
Fruit size	Tomato	signaling peptide gene	CRISPR-Cas9	2018	Rodríguez-Leal, D.; Lemmon, Z. H.; Man, J.; Bartlett, M. E.; Lippman, Z. B. Engineering quantitative trait variation for crop improvement by genome editing. Cell 2017, 171 (2), 470-480. e8.
	Tomato	an AP2/ERF transcription factor which regulates floral meristem activity	CRISPR-Cas9	2017	Yuste-Lisbona, F. J.; Fernández-Lozano, A.; Pineda, B.; Bretones, S.; OrtízAtienza, A.; García-Sogo, B.; Müller, N. A.; Angosto, T.; Capel, J.; Moreno, V. ENO regulates tomato fruit size through the floral meristem development network. Proc. Natl. Acad. Sci. U.S.A. 2020, 117 (14), 8187-8195

Grain Size	-	Negative regulator of mevalonic acid	CRISPR/Cas9	2018	Miao, C.; Wang, D.; He, R.; Liu, S.; Zhu, J. K. Mutations in MIR 396e and MIR 396f increase grain size and modulate shoot architecture in rice. Plant Biotechnol. J. 2020, 18 (2), 491-501.
	Rice	Negatively regulates the grain width and weight	CRISPR-Cas9	2020	Zhou, J.; Xin, X.; He, Y.; Chen, H.; Li, Q.; Tang, X.; Zhong, Z.; Deng, K.; Zheng, X.; Akher, S. A. Multiplex QTL editing of grain-related genes improves yield in elite rice varieties. Plant Cell Rep. 2019, 38 (4), 475-485.<br>Xu, R.; Yang, Y.; Qin, R.; Li, H.; Qiu, C.; Li, L.; Wei, P.; Yang, J. Rapid improvement of grain weight via highly efficient CRISPR/Cas9-mediated multiplex genome editing in rice. J. Genet. Genomics 2016, 43 (8), 529-532.
	Rice	Negatively regulates the grain size	CRISPR-Cas9	2020	Yuyu, C.; Aike, Z.; Pao, X.; Xiaoxia, W.; Yongrun, C.; Beifang, W.; Yue, Z.; Liaqat, S.; Shihua, C.; Liyong, C. Effects of GS3 and GL3. 1 for Grain Size Editing by CRISPR/Cas9 in Rice. Rice Sci. 2020, 27 (5), 405-413.
	Wheat	Negatively regulates the grain size	CRISPR-Cas9	2020	Ma, L.; Li, T.; Hao, C.; Wang, Y.; Chen, X.; Zhang, X. Ta GS 5-3A, a grain size gene selected during wheat improvement for larger kernel and yield. Plant Biotechnol. J. 2016, 14 (5), 1269-1280.
Nutrition quality	Camelina	Reduced oil content	CRISPR/Cas9 SDN1	2017	Aznar-Moreno JA, Durrett TP. Simultaneous Targeting of Multiple Gene Homeologs to Alter Seed Oil Production in Camelina sativa. Plant Cell Physiol. 2017;58:1260–7. doi:10.1093/pcp/pcx058.

Camelina	Higher oleic acid content, lower fatty acid content	CRISPR/Cas9 SDN1	2017	Jiang WZ, Henry IM, Lynagh PG, Comai L, Cahoon EB, Weeks DP. Significant enhancement of fatty acid composition in seeds of the allohexaploid, Camelina sativa, using CRISPR/Cas9 gene editing. Plant Biotechnol J. 2017;15:648–57. doi:10.1111/pbi.12663.
Camelina	Higher oleic acid content, lower fatty acid content	CRISPR/Cas9 SDN1	2017	Morineau C, Bellec Y, Tellier F, Gissot L, Kelemen Z, Nogué F, Faure J-D. Selective gene dosage by CRISPR-Cas9 genome editing in hexaploid Camelina sativa. Plant Biotechnol J. 2017;15:729–39. doi:10.1111/pbi.12671.
Camelina	Higher oleic acid and α linolenic acid content	CRISPR/Cas9 SDN1	2018	Ozseyhan ME, Kang J, Mu X, Lu C. Mutagenesis of the FAE1 genes significantly changes fatty acid composition in seeds of Camelina sativa. Plant Physiol Biochem. 2018;123:1–7. doi:10.1016/j.plaphy.2017.11.021.
Camelina	Higher oleic acid content, lower fatty acid content	CRISPR/Cas9 SDN1	2019	Rothamsted Research. 2019. https://gmoinfo.jrc.ec.europa.eu/gmp_report.aspx?CurNot=B/GB/19/R08/01. Accessed 28 Oct 2019.
Canola	Changed oil composition	CRISPR/Cas9 SDN1	2018	Okuzaki A, Ogawa T, Koizuka C, Kaneko K, Inaba M, Imamura J, Koizuka N. CRISPR/Cas9-mediated genome editing of the fatty acid desaturase 2 gene in Brassica napus. Plant Physiol Biochem 2018. doi:10.1016/j.plaphy.2018.04.025.
Cassava	modified starch composition	CRISPR/Cas9 SDN1	2018	Bull SE, Seung D, Chanez C, Mehta D, Kuon JE, Truernit E, et al. Accelerated ex situ breeding of GBSS- and PTST1-edited cassava for modified starch. Sci Adv. 2018;4.

Field pennycress	Changed oil composition	CRISPR/Cas9 SDN2	2019	McGinn M, Phippen WB, Chopra R, Bansal S, Jarvis BA, Phippen ME, et al. Molecular tools enabling pennycress (Thlaspi arvense) as a model plant and oilseed cash cover crop. 1467-7644. 2019;17:776–88. doi:10.1111/pbi.13014.
Lettuce	Reduced/ slowed browning of leaves	CRISPR/Cas9 SDN1	NA	Intrexon better DNA. APHIS-Am I Regulated. https://www.aphis.usda.gov/biotechnology/downloads/reg_loi/18-243- 01_a4_air_cbidel.pdf.
Maize	modified starch composition	CRISPR/Cas9 SDN1	2018	Qi X, Le Dong, Liu C, Mao L, Liu F, Zhang X, et al. Systematic identification of endogenous RNA polymerase III promoters for efficient RNA guide-based genome editing technologies in maize. The Crop Journal. 2018;6:314–20. doi:10.1016/j.cj.2018.02.005.
Maize	Waxycorn,	CRISPR/Cas9 SDN1	2015	United States Department of Agriculture (USDA). 2015. https://www.aphis.usda.gov/biotechnology/downloads/reg_loi/15-352- 01_air_inquiry_cbidel.pdf. Accessed 25 Aug 2018.
Millet	Reduction of lignin	CRISPR/Cas9 SDN1	2017	Park J-J, Yoo CG, Flanagan A, Pu Y, Debnath S, Ge Y, et al. Defined tetra-allelic gene disruption of the 4-coumarate:coenzyme A ligase 1 (Pv4CL1) gene by CRISPR/Cas9 in switchgrass results in lignin reduction and improved sugar release. Biotechnol Biofuels. 2017;10:284. doi:10.1186/s13068-017-0972-0.
Orchid	Reduction of Lignocellulose (lignification)	CRISPR/Cas9 SDN1	2016	Kui L, Chen H, Zhang W, He S, Xiong Z, Zhang Y, et al. Building a Genetic Manipulation Tool Box for Orchid Biology: Identification of Constitutive Promoters and Application of CRISPR/Cas9 in the Orchid, Dendrobium officinale. Front Plant Sci. 2016;7:2036. doi:10.3389/fpls.2016.02036.

peanut	Increased oleic acid content, reduced linoleic acid content	TALENs SDN1	2018	Wen SJ, Liu H, Li XY, Chen XP, Hong YB, Li HF, et al. TALEN-mediated targeted mutagenesis of fatty acid desaturase 2 (FAD2) in peanut (Arachis hypogaea L.) promotes the accumulation of oleic acid. Plant Mol Biol. 2018;97:177–85. doi:10.1007/s11103- 018-0731-z.29
Poppy	Reduced morphine and thebain content	CRISPR/Cas9 SDN1	2016	Alagoz Y, Gurkok T, Zhang B, Unver T. Manipulating the Biosynthesis of Bioactive Compound Alkaloids for Next-Generation Metabolic Engineering in Opium Poppy Using CRISPR-Cas 9 Genome Editing Technology. Sci Rep. 2016;6:30910–8. doi:10.1038/srep30910.
Potato	Improved starch quality	CRISPR/Cas9 SDN1	2017	Andersson M, Turesson H, Nicolia A, Fält A-S, Samuelsson M, Hofvander P. Efficient targeted multiallelic mutagenesis in tetraploid potato (Solanum tuberosum) by transient CRISPR-Cas9 expression in protoplasts. Plant Cell Rep. 2017;36:117–28. doi:10.1007/s00299-016-2062-3.
Potato	Improved starch quality	CRISPR/Cas9 SDN1	2018	Kusano H, Ohnuma M, Mutsuro-Aoki H, Asahi T, Ichinosawa D, Onodera H, et al. Establishment of a modified CRISPR/Cas9 system with increased mutagenesis frequency using the translational enhancer dMac3 and multiple guide RNAs in potato. Sci Rep-Uk. 2018;8.
Potato	Elimination of glycoalcaloides	CRISPR/Cas9 SDN1	2018	Nakayasu M, Akiyama R, Lee HJ, Osakabe K, Osakabe Y, Watanabe B, et al. Generation of α-solanine-free hairy roots of potato by CRISPR/Cas9 mediated genome editing of the St16DOX gene. Plant Physiol Biochem 2018. doi:10.1016/j.plaphy.2018.04.026.

Potato	Reduced glycoalcaloides	TALENs SDN1	2014	Sawai S, Ohyama K, Yasumoto S, Seki H, Sakuma T, Yamamoto T, et al. Sterol side chain reductase 2 is a key enzyme in the biosynthesis of cholesterol, the common precursor of toxic steroidal glycoalkaloids in potato. Plant Cell. 2014;26:3763–74. doi:10.1105/tpc.114.130096.
Potato	Reduced black spotting	TALENs SDN1	2016	United States Department of Agriculture (USDA). 2016. https://www.aphis.usda.gov/biotechnology/downloads/reg_loi/16-320-01_air_inquiry.pdf. Accessed 25 Aug 2018.
Potato	Improved starch quality	CRISPR/Cas9 SDN1	2019	Veillet F, Chauvin L, Kermarrec MP, Sevestre F, Merrer M, Terret Z, et al. The Solanum tuberosum GBSSI gene: a target for assessing gene and base editing in tetraploid potato 2019. doi:10.1007/s00299-019-02426-w.
Rice	Oleic acid contents	CRISPR/Cas9	2020	Abe, K.; Araki, E.; Suzuki, Y.; Toki, S.; Saika, H. Production of high oleic/low linoleic rice by genome editing. Plant Physiol. Biochem. 2018, 131, 58-62.
Rice	β-carotene content	CRISPR/Cas9	2019	Dong, O. X.; Yu, S.; Jain, R.; Zhang, N.; Duong, P. Q.; Butler, C.; Li, Y.; Lipzen, A.; Martin, J. A.; Barry, K. W. Marker-free carotenoid-enriched rice generated through targeted gene insertion using CRISPR-Cas9. Nat. Commun. 2020, 11 (1), 1-10.
Rice	Phytic acid synthesis	CRISPR/Cas9	2020	Khan, M. S. S.; Basnet, R.; Islam, S. A.; Shu, Q. Mutational analysis of OsPLDα1 reveals its involvement in phytic acid biosynthesis in rice grains. J. Agric. Food. Chem. 2019, 67 (41), 11436-11443.
Rice	Changed oil composition	CRISPR/Cas9 SDN1	2018	Abe K, Araki E, Suzuki Y, Toki S, SAIKA H. Production of high oleic/low linoleic rice by genome editing. Plant Physiol Biochem 2018. doi:10.1016/j.plaphy.2018.04.033.

Rice	Changed starch content (reduced amylose content)	CRISPR/Cas9 SDN2	2019	Fei YY, Yang J, Wang FQ, Fan FJ, Li WQ, Wang J, et al. Production of Two Elite Glutinous Rice Varieties by Editing Wx Gene. Rice Sci. 2019;26:118–24. doi:10.1016/j.rsci.2018.04.007
Rice	Changed starch content (reduced amylose content)	CRISPR/Cas9 SDN1	2018	Han Y, Luo DJ, Usman B, Nawaz G, Zhao N, Liu F, Li RB. Development of High Yielding Glutinous Cytoplasmic Male Sterile Rice (Oryza sativa L.) Lines through CRISPR/Cas9 Based Mutagenesis of Wx and TGW6 and Proteomic Analysis of Anther. Agronomy-Basel. 2018;8.
Rice	Reduction of ingredients harmful to health (caesium content)	CRISPR/Cas9 SDN1	2017	Nieves-Cordones M, Mohamed S, Tanoi K, Kobayashi NI, Takagi K, Vernet A, et al. Production of low-Cs+ rice plants by inactivation of the K+ transporter OsHAK1 with the CRISPR-Cas system. Plant J. 2017;92:43–56. doi:10.1111/tpj.13632.
Rice	Changed starch content (reduced amylose content)	CRISPR/Cas9 SDN1	2019	Pérez L, Soto E, Farré G, Juanos J, Villorbina G, Bassie L, et al. CRISPR/Cas9 mutations in the rice Waxy/GBSSI gene induce allele-specific and zygosity-dependent feedback effects on endosperm starch biosynthesis 2019. doi:10.1007/s00299-019-02388-z.
Rice	Scented rice	TALENs SDN1	2015	Shan Q, Zhang Y, Chen K, Zhang K, Gao C. Creation of fragrant rice by targeted knockout of the OsBADH2 gene using TALEN technology. Plant Biotechnol J. 2015;13:791–800. doi:10.1111/pbi.12312.
Rice	Scented rice	CRISPR/Cas9 SDN1	2017	Shen L, Hua Y, Fu Y, Li J, Liu Q, Jiao X, et al. Rapid generation of genetic diversity by multiplex CRISPR/Cas9 genome editing in rice. Sci China Life Sci. 2017;60:506–15. doi:10.1007/s11427-017-9008-8.

Rice	Changed starch content (increased amylose content)	CRISPR/Cas9 SDN1	2017	Sun Y, Jiao G, Liu Z, Zhang X, Li J, Guo X, et al. Generation of High-Amylose Rice through CRISPR/Cas9-Mediated Targeted Mutagenesis of Starch Branching Enzymes. Front Plant Sci. 2017;8:1–15. doi:10.3389/fpls.2017.00298.
Rice	Reduction of ingredients harmful to health (cadmium content)	CRISPR/Cas9 SDN1	2017	Tang L, Mao B, Li Y, Lv Q, Zhang L, Chen C, et al. Knockout of OsNramp5 using the CRISPR/Cas9 system produces low Cd-accumulating indica rice without compromising yield. Sci Rep. 2017;7:14438. doi:10.1038/s41598-017-14832-9.
Rice		CRISPR/Cas9 SDN1	2017	Ye Y, Li P, Xu T, Zeng L, Cheng D, Yang M, et al. OsPT4 Contributes to Arsenate Uptake and Transport in Rice. Front. Plant Sci. 2017;8:311. doi:10.3389/fpls.2017.02197.
Rice	Waxy rice	CRISPR/Cas9 SDN1	2018	Zhang J, Zhang H, Botella JR, Zhu J-K. Generation of new glutinous rice by CRISPR/Cas9-targeted mutagenesis of the Waxy gene in elite rice varieties. J Integr Plant Biol. 2018;60:369–75. doi:10.1111/jipb.12620.
Soybean	High oleic acid content, low linoleic acid content	CRISPR/Cas9 SDN1	2019	Al Amin N, Ahmad N, Wu N, Pu X, Ma T, Du Y, et al. CRISPR-Cas9 mediated targeted disruption of FAD2-2 microsomal omega-6 desaturase in soybean (Glycine max.L) 2019. doi:10.1186/s12896-019-0501-2.
Soybean	High oleic acid content, low linoleic acid content	TALENs SDN2	2014	Haun W, Coffman A, Clasen BM, Demorest ZL, Lowy A, Ray E, et al. Improved soybean oil quality by targeted mutagenesis of the fatty acid desaturase 2 gene family. Plant Biotechnol J. 2014;12:934–40. doi:10.1111/pbi.12201

Soybean	High oleic acid content, low linoleic acid content	TALENs SDN1	2014	United States Department of Agriculture (USDA). 2014. https://www.aphis.usda.gov/biotechnology/downloads/reg_loi/cellectis_air_fad2k0_soy_c bidel.pdf. Accessed 25 Aug 2018.31
Sugar cane	Reduction of lignin	TALENs SDN1	2016	Jung JH, Altpeter F. TALEN mediated targeted mutagenesis of the caffeic acid Omethyltransferase in highly polyploid sugarcane improves cell wall composition for production of bioethanol. Plant Mol Biol. 2016;92:131–42. doi:10.1007/s11103-016- 0499-y.
Sugar cane	Reduction of lignin	TALENs SDN2	2018	Kannan B, Jung JH, Moxley GW, Lee S-M, Altpeter F. TALEN-mediated targeted mutagenesis of more than 100 COMT copies/alleles in highly polyploid sugarcane improves saccharification efficiency without compromising biomass yield. Plant Biotechnol J. 2018;16:856–66. doi:10.1111/pbi.12833
Tobacco	Reduced nicotine content	CRISPR/Cas9 SDN1	2019	Schachtsiek J, Stehle F. Nicotine-free, Non-transgenic Tobacco (Nicotianatabacuml.) Edited by CRISPR-Cas9. 1467-7644 2019. doi:10.1111/pbi.13193.
Tomato	Seedless fruits	CRISPR/Cas9 SDN1	2017	Klap C, Yeshayahou E, Bolger AM, Arazi T, Gupta SK, Shabtai S, et al. Tomato facultative parthenocarpy results from SlAGAMOUS-LIKE 6 loss of function. Plant Biotechnol J. 2017;15:634–47. doi:10.1111/pbi.12662.
Tomato	Increase of health-promoting ingredients (increased GABA content)	CRISPR/Cas9 SDN1	2018	Lee J, Nonaka S, Takayama M, Ezura H. Utilization of a Genome-Edited Tomato (Solanum lycopersicum) with High Gamma Aminobutyric Acid Content in Hybrid Breeding. J. Agric. Food Chem. 2018;66:963–71. doi:10.1021/acs.jafc.7b05171.

Tomato	Increase of health-promoting ingredients (increased lycopene content)	CRISPR/Cas9 SDN1	2018	Li X, Wang Y, Chen S, Tian H, Fu D, Zhu B, et al. Lycopene Is Enriched in Tomato Fruit by CRISPR/Cas9-Mediated Multiplex Genome Editing. Front. Plant Sci. 2018;9:179. doi:10.3389/fpls.2018.00559.
Tomato	Increase of health-promoting ingredients (increased GABA content)	CRISPR/Cas9 SDN1	2017	Nonaka S, Arai C, Takayama M, Matsukura C, Ezura H. Efficient increase of ɣaminobutyric acid (GABA) content in tomato fruits by targeted mutagenesis. Sci Rep. 2017;7:7057. doi:10.1038/s41598-017-06400-y.
Tomato	Longer storage at room temperature	CRISPR/Cas9 SDN1	2017	Yu Q-h, Wang B, Li N, Tang Y, Yang S, Yang T, et al. CRISPR/Cas9-induced Targeted Mutagenesis and Gene Replacement to Generate Long-shelf Life Tomato Lines. Sci Rep. 2017;7:818. doi:10.1038/s41598-017-12262-1.
Wheat	Longer seed dormancy (prevents germination before harvest)	CRISPR/Cas9 SDN1	2019	Abe F, Haque E, Hisano H, Tanaka T, Kamiya Y, Mikami M, et al. Genome-Edited Triple-Recessive Mutation Alters Seed Dormancy in Wheat. Cell Rep. 2019;28:1362- 1369.e4. doi:10.1016/j.celrep.2019.06.090.
Wheat (Durum)	Reduced gluten content	CRISPR/Cas9 SDN1	2018	Sánchez-León S, Gil-Humanes J, Ozuna CV, Giménez MJ, Sousa C, Voytas DF, Barro F. Low-gluten, nontransgenic wheat engineered with CRISPR/Cas9 2018. doi:10.1111/pbi.12837.

Product quality, Herbicide tolerance	Maize	reduced Phytat-production, Herbicide tolerance	ZFN SDN4	2009	Shukla VK, Doyon Y, Miller JC, DeKelver RC, Moehle EA, Worden SE, et al. Precise genome modification in the crop species Zea mays using zinc-finger nucleases. Nature. 2009;459:437–41. doi:10.1038/nature07992.
	Maize	reduced Phytat-production, Herbicide tolerance	ZFN SDN3	2010	United States Department of Agriculture (USDA). 2010. https://www.aphis.usda.gov/biotechnology/downloads/reg_loi/DOW_Email_%20to_Susa n_%20Kohler_032010.pdf. Accessed 25 Aug 2018.
Starch quality	Sweet potato	Reduce starch quality	CRISPR/Cas9	2017	Wang, H.; Wu, Y.; Zhang, Y.; Yang, J.; Fan, W.; Zhang, H.; Zhao, S.; Yuan, L.; Zhang, P. CRISPR/Cas9-based mutagenesis of starch biosynthetic genes in sweet potato (Ipomoea Batatas) for the improvement of starch quality. Int. J. Mol. Sci. 2019, 20 (19),
Storage performance	Potato	Improved cold storage and frying conditions (reduced sugar/ reduced acrylamide)	TALENs SDN1	2016	Clasen BM, Stoddard TJ, Luo S, Demorest ZL, Li J, Cedrone F, et al. Improving cold storage and processing traits in potato through targeted gene knockout. Plant Biotechnol J. 2016;14:169–76. doi:10.1111/pbi.12370.
	Rice	Longevity of seeds	TALENs SDN1	2015	Ma L, Zhu F, Li Z, Zhang J, Li X, Dong J, Wang T. TALEN-Based Mutagenesis of Lipoxygenase LOX3 Enhances the Storage Tolerance of Rice (Oryza sativa) Seeds. PLoS ONE. 2015;10:e0143877. doi:10.1371/journal.pone.0143877.

wheat allergies (bakers' asthma)	Wheat	wheat allergies (bakers' asthma)	CRISPR/Cas9	2017	Camerlengo, F.; Frittelli, A.; Sparks, C.; Doherty, A.; Martignago, D.; Larré, C.; Lupi, R.; Sestili, F.; Masci, S. CRISPR-Cas9 multiplex editing of the α-amylase/trypsin inhibitor genes to reduce allergen proteins in durum wheat. Front. sustain. food syst. 2020, 4, 104
